# Kronos: a workflow assembler for genome analytics and informatics

**DOI:** 10.1093/gigascience/gix042

**Published:** 2017-06-26

**Authors:** M. Jafar Taghiyar, Jamie Rosner, Diljot Grewal, Bruno M. Grande, Radhouane Aniba, Jasleen Grewal, Paul C. Boutros, Ryan D. Morin, Ali Bashashati, Sohrab P. Shah

**Affiliations:** 1 Department of Molecular Oncology, British Columbia Cancer Agency, 675 West 10th Ave, V5Z 1L3 Vancouver, BC, Canada; 2 Department of Pathology and Laboratory Medicine, University of British Columbia, 2211 Wesbrook Mall, V6T 2B5 Vancouver, BC, Canada; 3 Department of Molecular Biology and Biochemistry, Simon Fraser University, 8888 University Drive, V5A 1S6 Burnaby, BC, Canada; 4 Ontario Institute for Cancer Research (OICR), 661 University Avenue, M5G 0A3 Toronto, ON, Canada; 5 Department of Medical Biophysics, University of Toronto, 101 College Street, M5G 1L7 Toronto, ON, Canada

**Keywords:** genomics, workflow, pipeline, reproducibility

## Abstract

**Background:**

The field of next-generation sequencing informatics has matured to a point where algorithmic advances in sequence alignment and individual feature detection methods have stabilized. Practical and robust implementation of complex analytical workflows (where such tools are structured into “best practices” for automated analysis of next-generation sequencing datasets) still requires significant programming investment and expertise.

**Results:**

We present Kronos, a software platform for facilitating the development and execution of modular, auditable, and distributable bioinformatics workflows. Kronos obviates the need for explicit coding of workflows by compiling a text configuration file into executable Python applications. Making analysis modules would still require programming. The framework of each workflow includes a run manager to execute the encoded workflows locally (or on a cluster or cloud), parallelize tasks, and log all runtime events. The resulting workflows are highly modular and configurable by construction, facilitating flexible and extensible meta-applications that can be modified easily through configuration file editing. The workflows are fully encoded for ease of distribution and can be instantiated on external systems, a step toward reproducible research and comparative analyses. We introduce a framework for building Kronos components that function as shareable, modular nodes in Kronos workflows.

**Conclusions:**

The Kronos platform provides a standard framework for developers to implement custom tools, reuse existing tools, and contribute to the community at large. Kronos is shipped with both Docker and Amazon Web Services Machine Images. It is free, open source, and available through the Python Package Index and at https://github.com/jtaghiyar/kronos.

## Background

The emergence of next-generation sequencing (NGS) technology has created unprecedented opportunities to identify and study the impact of genomic aberrations on genome-wide scales. Data generation technology for NGS is stabilizing, and exponential declines in cost have made sequencing accessible to most research and clinical groups. Alongside progress in data generation capacity, a myriad of analytical approaches and software tools have been developed to identify and interpret relevant biological features. These include computational methods for raw data preprocessing, sequence alignment and assembly, variant identification, and variant annotation. However, major challenges are induced by rapid development and improvement of analytical methods. This makes construction of analytical workflows a near dynamic process, creating a roadblock to seamless implementation of linked processes that navigate from raw input to annotated variants.

As a consequence, robust analysis and continuous iterative improvements in the analysis of large sets of sequencing data remain labor intensive and costly and require considerable analytical expertise. As best practices (e.g., [1]) remain a moving target, software systems that can rapidly adapt to new (and optimal) solutions for domain-specific problems are necessary to facilitate high-throughput comparisons.

Several tools and frameworks for NGS data analysis and workflow management have been developed to address these needs. Galaxy [2] is an open, web-based platform to perform, reproduce, and share analyses. Using the Galaxy user interface, users can build analysis workflows from a collection of tools available through the Galaxy Tool Shed [3]. The Taverna suite [4] allows the execution of workflows that typically mix web services and local tools. Tight integration with myExperiment [5] gives Taverna access to a network of shared workflows, including NGS data processing.

Although the current workflow management systems such as Galaxy are great for routine bioinformatics tasks, the development of customized tools and workflows is not convenient, and experienced bioinformaticians commonly work at a lower programming level and write their own workflows in scripting languages such as Bash, Perl, or Python [6]. A number of lightweight workflow management tools have been specifically developed to simplify scripting for these target users, including Ruffus [7], Bpipe [8], and Snakemake [9]. Common Workflow Language [10] is another similar tool that has roots in GNU make and aims to build portable workflows across a variety of platforms by using a set of standard specification to define wrappers around command line tools as well as creating nested workflows. While these workflow management tools reduce development overhead, users still need to write a substantial amount of routine code to create their own workflows, maintain the existing ones, replace subsets of workflows with new ones, and run subsets of existing workflows.

To further facilitate the process of creating workflows, Omics-Pipe proposed a framework to automate best practice multi-omics data analysis workflows based on Ruffus [11]. It offers several preexisting workflows and reduces the development overhead for tracking the run of each workflow and logging the progress of each analysis step. However, it remains cumbersome to create a custom workflow with Omics-Pipe as users need to manually write a Python script for the new workflow by copy/pasting a specific header to the script and writing the analysis functions using Ruffus decorators. The same applies when adding or removing an analysis step to an existing workflow.

We introduce a highly flexible open source Python-based software tool (Kronos) that enables bioinformatics developers, i.e., bioinformaticians who develop workflows for analyzing genomic data, to quickly create a workflow. It uses Ruffus [7] as the underlying workflow management system and adds a level of abstraction on top of it, which significantly reduces programming overhead for workflow development and provides a mechanism to represent a workflow by a top-level YAML configuration file.

Kronos is shipped with Docker and Amazon Machine Images to further facilitate its use locally on high performance computing clusters and in the cloud infrastructures. A number of workflows for the analysis of single human genomes and cancer tumour-normal pairs following best analysis practices accompany Kronos and are freely available. 

## Results

**Figure 1:**
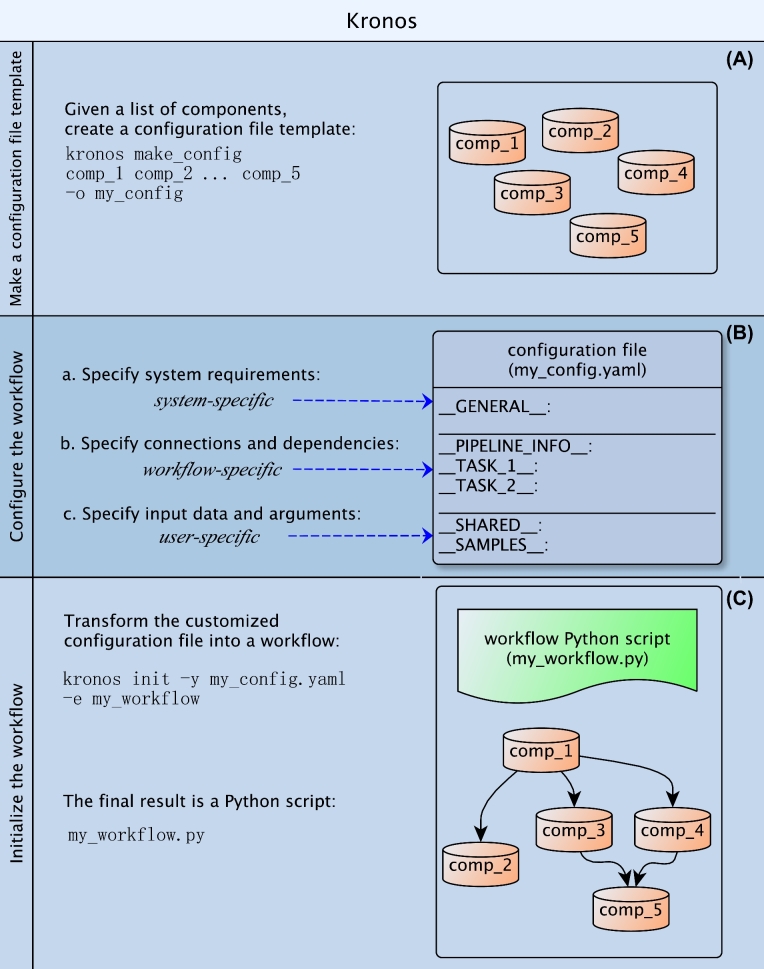
Make a workflow. Making a new workflow with Kronos includes 3 steps: (A) make a configuration file template: given a set of existing components, users can generate this file by running the command make_config; (B) configure the workflow: users can specify the desirable flow of their workflow using the connections and dependencies, customize output directory names, and specify input arguments and data to the required fields in the configuration file template; (C) initialize the workflow: this is achieved by running the command init on the configuration file, which transforms the YAML file into the Python workflow script.

Kronos creates modular workflows that can be easily updated by editing their corresponding configuration file. Each module in the workflow corresponds to a component, which is a wrapped command line tool (i.e., described in more detail later). As shown in Fig. 1, users can create a workflow from a set of existing components by following the 3 steps listed below (referred to as Steps 1, 2, and 3 in the remainder of this paper). Section 2 of Additional file 1 provides an example of how to make a variant calling workflow.

- Step 1. Given a set of existing components, create a configuration file template by running the following Kronos command:
   kronos make_config
   [list of components] -o <output_name>
  where [list of components] refers to the component names that we aim at using in our workflow.
- Step 2. In the configuration file template, specify the order by which the components in the workflow should be run. This does not require programming skills and is merely text-based.
- Step 3. Create the workflow by running the following Kronos command with the configuration file as its input:
   kronos init -y <config_file.yaml>
   -o <workflow_name>

The output is an executable Python script that runs the workflow. Depending on its corresponding configuration file, the script is encoded to automatically parallelize eligible tasks, provide pause/resume functionality, make unique run IDs, make the desired output directory tree, submit jobs to cluster or run them locally, and log the events.

### Kronos components

A component is a wrapper around a command line tool that encapsulates all the required programming. The purpose of components is to modularize workflows with reusable building blocks that require minimal development. As shown in Section 1 of Additional file 1, the number of lines of codes for making a new component is very small. The simple development instructions eliminate, e.g., the need to use Ruffus decorators, input/output management using regex expressions, and complicated dependency management in the code that can easily become very complex with the number of tasks in a workflow. Furthermore, a large workflow can be divided into a set of small components that results in a much faster and more manageable workflow development. Kronos also provides a command for making component templates that helps develop a new component in a few minutes.

All command line tools, such as a simple copy command or a complicated single nucleotide variant (SNV) caller, can be wrapped as Kronos components. Regardless of how complicated they are, their corresponding components have a standard directory structure composed of specific wrappers and subdirectories. The wrappers are also independent of the programming language used for developing the command line tool.

The components should be developed prior to making the workflow. However, since they are individually and independently developed and due to their reusability, the initial preparation of a component happens only once, and various workflows can use the already developed component.

**Figure 2:**
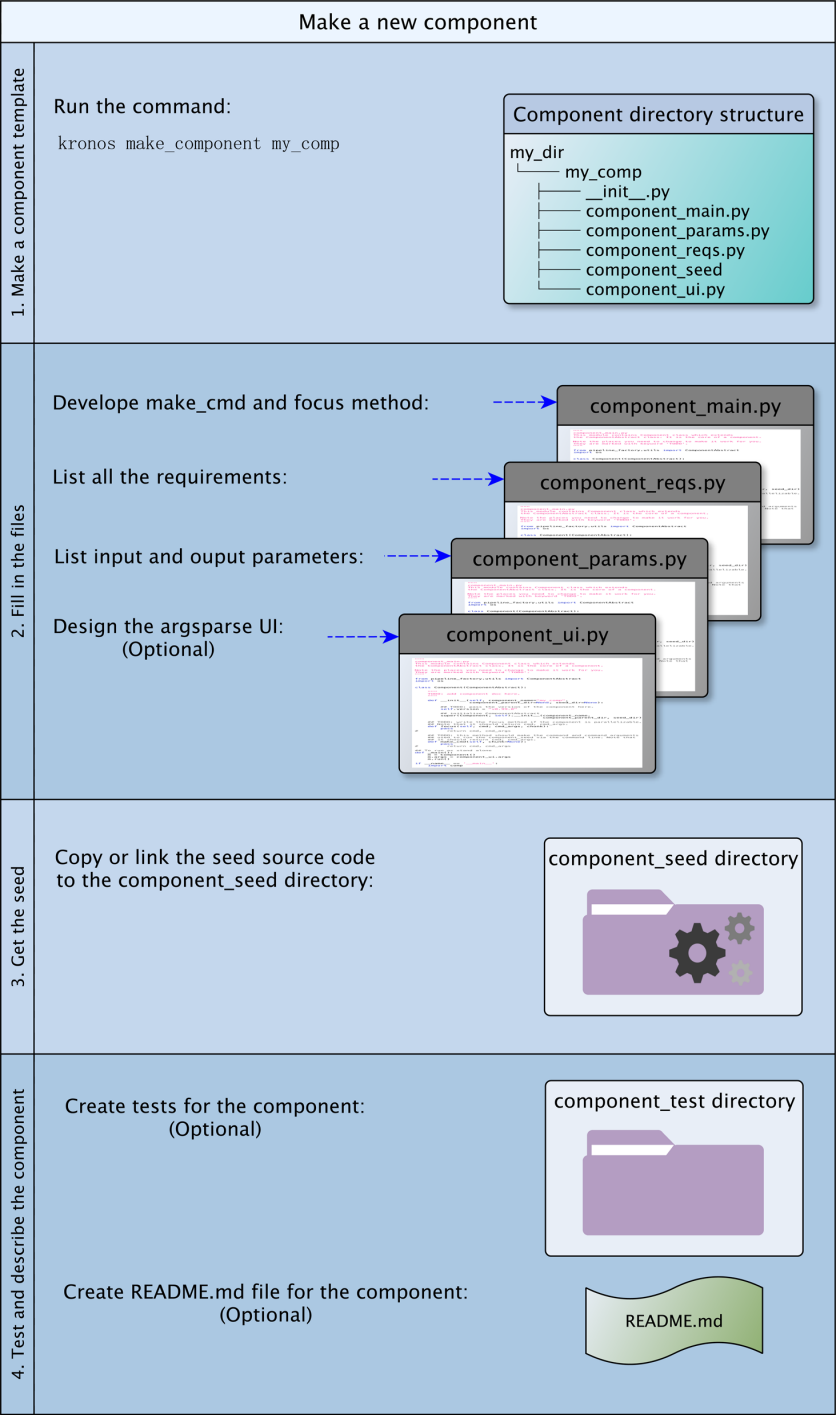
Make a component. Making a new component for Kronos includes the following steps: (A1) make a new component template by running the command make_component; (B2) fill in the resulting template accordingly; (C3) copy or link the source code of the *seed* used in the component; (D4) optionally create README.md and tests for the component.

### Kronos configuration file

Kronos workflows are represented by a YAML configuration file. For a given set of components, the Kronos make_config command generates a configuration file template that is mostly prefilled with default values. For each input component, there is a corresponding section with a unique name in the configuration file called task. Users should use these sections to specify the order by which each task in the workflow should be run (Step 2 of creating a workflow). This can be done by a simple convention called IO-connection. An IO-connection is basically a pair of values comprising a task name and 1 of its parameters. It determines which task should be followed by the current task and is specified as an argument to 1 of the parameters of the current task. For example, in the following configuration file, (’__TASK_1__’, ’out_file’) is an IO-connection that makes __TASK_2__ follow __TASK_1__, i.e., the input to the parameter in_file of __TASK_2__ comes from the parameter out_file of __TASK_1__.

__TASK_1__:

 out_file: ’foo.txt’

__TASK_2__:

 in_file: (’__TASK_1__’, ’out_file’)

The run options for each task are also set in the configuration file, including granular resource requests such as free memory or the number of CPUs, running locally or on cluster, running with parallelization, pause/resume functionality, etc.

A configuration file has the following blocks (see Additional file 1: Fig. S1):

- system-specific, which captures the system-dependant requirements of the workflow (such as the paths to the local installations) and includes the GENERAL and PIPELINE_INFO sections;
- user-specific, which contains the input files and arguments and includes the SHARED and SAMPLES sections
- workflow-specific, which defines the connection between the components in the workflow. Task sections related to each component are in this group.

This design has the following advantages: (i) if users want to rerun the same workflow for various sets of input files and arguments, they would only need to update the user-specific sections. This prevents inadvertent changes in the flow of the workflow when changing the inputs; and (ii) the segregation of system-specific information from the rest of the sections enables users to run a workflow practically anywhere. In other words, by simply updating the system-specific sections with proper values, the requirements of the workflow can be observed on any machine.

### Kronos workflows

**Figure 3:**
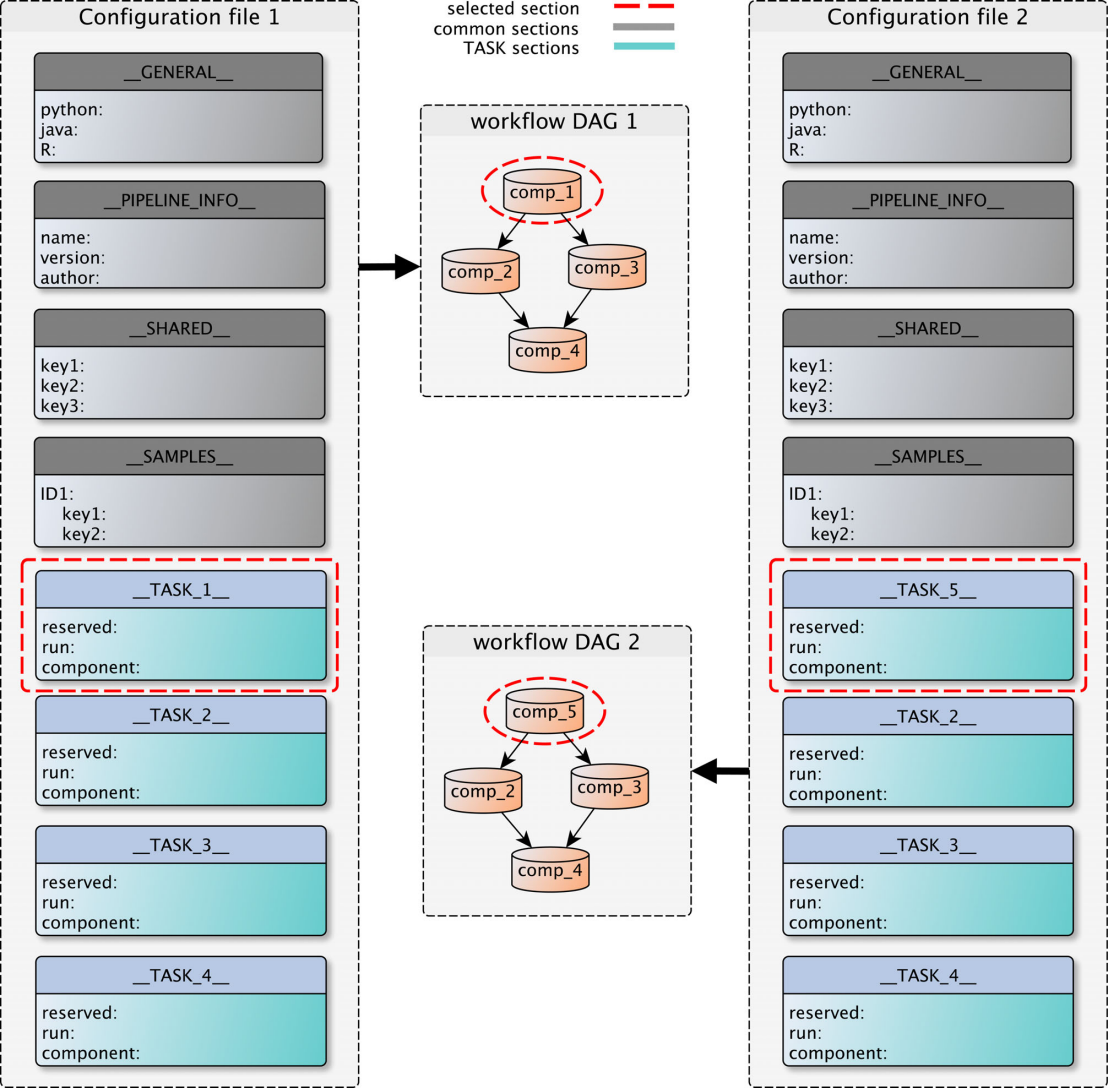
Replace a component in a workflow. The configuration file has different sections as shown in the figure. These sections are: GENERAL, PIPELINE_INFO, SHARED, SAMPLES, and TASKs. The modular organization of the configuration file allows for easy customization of workflows, which can serve different purposes such as tool comparison. Adding, removing, or replacing nodes in the DAG of the workflows can be easily done by adding, removing, or replacing the corresponding TASK sections in the configuration file. For instance, to go from workflow DAG1 to workflow DAG2, i.e., to replace comp_1 (e.g., variant caller 1) in the first workflow with comp_5 (e.g., variant caller 2) in the second, the user only needs to replace the TASK_1 section with the TASK_5 section in the configuration file and perform Step 3.

**Figure 4:**
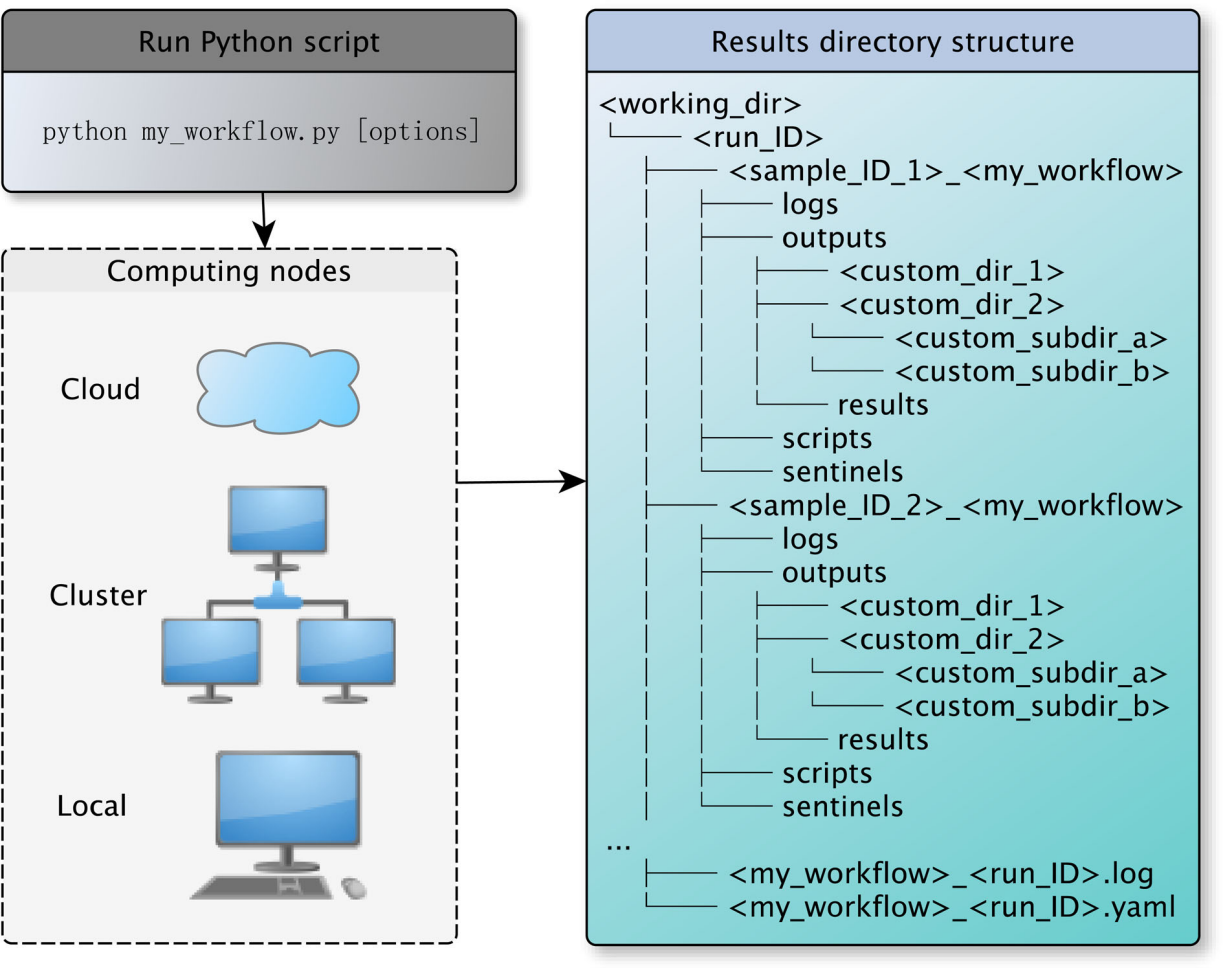
Run a workflow. Workflows generated by Kronos are ready to run locally on a cluster of computing nodes and in the cloud. To run a workflow, users only need to run the Python workflow script. Each run of a workflow generates a specific directory structure tagged with a run-ID. When running a workflow for multiple samples, a separate directory is made for each sample to make it convenient to locate the results corresponding to each sample. This figure shows the tree structure of the resulting directory. There are 4 subdirectories that are always generated for each sample: (Ai) logs: to store the log files; (Bii) outputs: to store all the output files generated by all the components in the workflow; (iii) scripts: to store the scripts automatically generated by Kronos to run each component in the workflow; (iv) sentinels: to store sentinel files used by Kronos to pick up the workflow from where it left off in a previous run.

Each workflow made by Kronos is a directed acyclic graph (DAG) of components where every node in the graph corresponds to a task section in the configuration file. Task sections can independently be added, removed, or replaced in the configuration file (Fig. 3). Therefore, to add, remove, or replace a component in the workflow or equivalently a node in the DAG, users simply need to change the corresponding task section in the configuration file and run the command in Step 3. As a result, the workflows are highly modular and maintaining them is as easy as updating the configuration file without having to rewrite the workflow. Finally, a workflow can be run by simply running the Python workflow script using the command line as depicted in Fig. 4.

### Kronos features and benefits

Full details of how to use each of the following features can be found in the software documentation.

#### Parameter sweeping

It is sometimes desired to run a particular tool or algorithm with various sets of parameters in order to select the parameter set with superior performance for a given problem. For example, a user may want to find the proper model parameters (such as mapping quality and base quality thresholds) for a variant calling tool to accurately detect single nucleotide variants. Kronos provides a mechanism for this purpose where users can specify all different sets of input arguments (or parameters) in the SAMPLES section of the configuration file. In this case, running Step 3 creates a number of intermediate workflows, each for 1 set of input arguments, along with the main workflow. When running the main workflow, Kronos runs the intermediate workflows in parallel, each on one set of the input arguments. We have provided a variant calling workflow with parameter sweeping functionality in Section 3 of Additional file 1 to demonstrate this feature.

#### Tool comparison

In bioinformatics, it is often required to compare the performance of 2 or more algorithms or compare a new analysis tool to the existing ones to select the 1 that best fits the particular goals of a project. For example, it is often helpful to evaluate the performance of different variant calling algorithms [12]. The modularity of the workflows generated by Kronos facilitates the comparison of different algorithms and tools. For this purpose, as shown in Fig. 3, the user can simply replace a task section corresponding to an analysis tool with another task section corresponding to another similar tool and run Step 3.

#### Automatic parallelization and merge

Most of the recent tools developed in the bioinformatics field are parallelizable or have the potential to run in parallel. However, the majority of these tools are shipped without the built-in functionality and require the users to manually break the analysis into smaller analyses. For example, many variant calling algorithms are capable of running on user-specified coordinates of the genome but are not shipped with parallelization functionality. However, a user can analyze whole genome sequencing data chunk by chunk in parallel with the caveat of manually scripting the parallelization steps. Due to the cumbersome nature of manual parallelization, many users might avoid running the tools in parallel, which considerably increases the runtime of the analysis. To resolve this issue, Kronos automatically parallelizes tasks in the workflow if feasible. Then, it aggregates the outputs of all child tasks and merges them if necessary.

#### Reproducible workflows

The configuration file and components of a workflow are portable.

**Figure 5:**
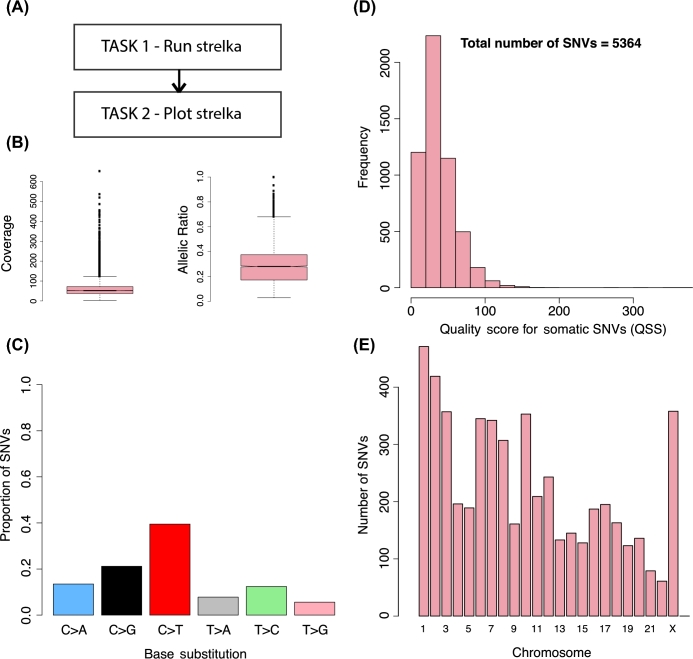
Strelka workflow. Results from the tumour-normal variant calling workflow on whole genome data of a breast cancer case (SA500 - EGA accession number EGAS00001000952). (A) Schematic of the workflow, which is comprised of 2 tasks. The plots generated by the workflow are in fact the output of TASK_2: (B) box plot of coverage and variant allelic ratios for the SNVs detected by Strelka, (C) base substitution patterns for the somatic SNVs, and (D) total number of SNVs and their histogram based on the quality score (QSS), (E) distribution of the number of SNVs across different chromosomes.

Therefore, users can readily duplicate a workflow elsewhere by only running the kronos init command in Step 3. To show this functionality, we have included an example of a workflow that performs somatic variant calling on whole genome data of a breast cancer case using the Strelka algorithm [13] and generates a number of plots based on Strelka calls (Fig. 5). Detailed step-by-step instructions to reproduce this figure are in Section 3 of Additional file 1. It should be noted that Kronos workflows can be duplicated elsewhere but the user would still need to manage tool installations and dependencies.

#### Cloud support

The massive scale of genomic data justifies a move to the cloud for storage and analyses in order to minimize cost and handle the ebb and flow of computational demands. Kronos’ flexibility addresses the emerging need for rapid deployment of analysis workflows in the cloud. Several command line tools exist for managing fleets of compute nodes on cloud platforms such as Amazon Web Services, including StarCluster, CfnCluster, and Elasticluster. A guide on the creation and management of a cloud cluster using StarCluster software and deployment of Kronos is provided in the online documentation, and an Amazon Machine Image is provided for convenience.

#### Controlled pause/resume by breakpoints

When running a workflow, certain blocks of the workflow may need to run multiple times, e.g., to tune a particular parameter of a component or to inspect the results of the previous tasks in the workflow before the next tasks are triggered. Analogous to the debuggers, Kronos provides users with breakpoints to perform a controlled pause/resume action.

In addition, with the breakpoint mechanism, users can break the flow of a workflow into several subworkflows and run each part on a different machine or cluster. In other words, once a breakpoint happens, i.e., 1 subworkflow is complete, the main workflow can be transferred to a different machine and it will pick up running from where it left off on the previous machine, provided that all the intermediate files are present. For example, a workflow can contain a component as its last step that loads the final results to a local database that can be reached only from a specific IP or machine. In this case, the user can run the workflow on a powerful computing node or a cluster with a breakpoint set for the component prior to the last component, i.e., database loader in this example. Once the breakpoint is applied, the user can resume the workflow on the other machine, so that the results can be loaded to the local database.

#### Forced dependency

Often in a workflow, a task requires the output of the previous one. As explained earlier, Kronos handles this explicit dependency by IO-connection. However, sometimes a task might need to wait for 1 one or more other steps in the workflow to finish although there are no explicit IO-connections between them. For example, when 2 tasks intend to write results in the same file, one needs to make sure that both tasks do not run at the same time. Another example would be a variant calling algorithm (e.g., GATK) that accepts a bam file as input. However, it also expects the index of the bam file to be present in the same directory as the bam file. If the index is created in 1 of the previous tasks in the workflow, then the current task that needs the bam file and its index would depend implicitly on the other task that creates the index file. In this case, a mechanism is required to force the variant calling task to wait until the index file is ready. Kronos provides a forced dependency feature to overcome this problem (see Additional file 1: Fig. S2).

#### Results directory customization

It is desirable to have full control of the structure of the results directory when running a workflow. With Kronos, users can readily determine the structure of the results directory in the configuration file. This provides easy file management for the users. Figure 4 shows an example of the tree structure of the results directory generated for a workflow.

#### Boilerplates

Users can use this feature to insert a command or a script into the begining of the command used to run a task in a workflow. This is particularly useful for setting up the environments using the Environment Modules package [14]. It also provides a means to run preprocessing steps for a specific task prior to running the task itself.

#### Keywords

There are several specific keywords that users can use in the configuration file that will be automatically replaced by proper values in runtime. This enables users to customize the paths and file names based on the workflow-specific values in runtime such as run-ID, workflow name, or sample ID.

## Workflows

We have developed a number of standard genome analysis workflows using Kronos. These workflows utilize many of the Kronos features introduced earlier and are publicly available.

## 

### Alignment workflow

This workflow accepts paired-end FASTQ files as input and aligns them using the Burrows-Wheeler aligner [15]. It also sorts the aligned bam file, flags the duplicates, indexes the file, and generates statistics for the final bam file.

## 

### Germline variant calling workflow

This workflow is an implementation of the best practices guide established by the Broad Institute [1] applied to variant discovery using haplotypecaller. In short, it runs the Bowtie2 aligner, creates targets using GATK RealignerTargetCreator, and calls SNVs and indels using GATK.

## 

### Copy number estimation workflow

HMMCopy is a suite of tools for copy number estimation of whole genome sequencing data [16]. This workflow takes a bam file as an input and estimates the copy number with GC and mappability correction using HMMCopy. It also segments and classifies the copy number profiles with a robust Hidden Markov Model.

## 

### Somatic variant calling workflow

This workflow takes a pair of tumour/normal bam files as inputs and detects the somatic SNVs and indels using the Strelka algorithm [13], annotates the resulting VCF files using SnpEff [17], and flags the variants observed in 1000 genomes and dbSNP databases.

## 

### RNA-seq analysis workflow

This workflow aligns RNA-seq FASTQ files using STAR aligner [18], followed by Cufflinks, which assembles transcriptomes from RNA-seq data and quantifies their expression [19].

## Conclusions

A foundation for rapid and reliable implementation of genomic analysis workflows is an essential need as a myriad of potential applications of genomics (ranging from personalized cancer therapies to monitoring the evolution and spread of infectious diseases) are projected to produce massive amounts of genomic data in the next few years. We have developed Kronos to address this need by expediting workflow development. It minimizes the tedious process of writing code by transforming a YAML configuration file into a Python script and manages its execution. Given a set of premade components, constructing a workflow by Kronos does not need programming skills as the user only needs to fill out specific sections of the configuration file. Making components still requires programming. However, their development time and effort is minimal given their design structure. They also provide a powerful and highly flexible framework for bioinformatics developers to fully customize their workflows with reusable modules.

A number of standard genomic analysis workflows and their building components that have been made by Kronos accompany this software and are available to the public. Kronos has been developed for genomics applications, but it can be readily utilized in other scientific and nonscientific fields.

The configuration file and components of a Kronos workflow are portable. This is a step toward reproducible research; however, it should be noted that while Kronos workflows can be duplicated elsewhere, the user would still need to manage tool installations and dependencies. For fully reproducible research, a Docker image of the whole workflow or the environment is perhaps more plausible. Kronos is complementary to other efforts for reproducible research. For example, in order to unify representation of workflow definitions and tool wrappers, the Common Workflow Language working group [10] and the Workfow Description Language [20] offer specifications that enable data scientists to describe analysis tools and workflows that are human-readable, easy to use, portable, and support reproducibility. It would be beneficial for workflow management tools to adopt these representation standards once they are agreed upon in the field.

In conclusion, this work provides a framework toward rapid integration of new (and optimal) genomic analysis advances in high-throughput studies. The flexibility, customization, and modularity of Kronos make it an attractive system to use in any high-throughput genomics analysis endeavour. We expect that Kronos will provide a foundational platform to accelerate toward the need to standardize and distribute NGS workflows in both clinical and research applications.

## Additional files

### Additional file 1 — Supplementary information

The supplementary information is in pdf format and expalins (a) how to make a component, (b) how to make a workflow, (c) how to run a workflow, and (d) Fig. S1 and Fig. S2.

## Abbreviations

DAG: directed acyclic graph; NGS: next-generation sequencing; SNV: single nucleotide variant.

## Availability and requirements

Project name: Kronos

Project home page: https://github.com/jtaghiyar/kronos

Operating system(s): Linux, Windows, Mac OS

Programming language: Python 2.7.5

Other requirements: Ruffus, PyYaml

License: MIT

## Availability of supporting data

Snapshots of the code can be found in the *GigaScience* repository, GigaDB [21].

## Supplementary Material

GIGA-D-17-00052_Original-Submission.pdf

GIGA-D-17-00052_Revision-1.pdf

Response-to-Reviewer-Comments_Original-Submission.pdf

Reviewer-1-Report-(Original-Submission).pdf

Supplementary Information
