# Supplementary material for: Kronos: a workflow assembler for genome analytics and informatics
Source: Gigascience. 2017 Jun 26;6(7):1–10. doi: 10.1093/gigascience/gix042 (PMC5569921; doi:10.1093/gigascience/gix042)
Supplement: GIGA-D-17-00052_Original-Submission.pdf [file gix042_GIGA-D-17-00052_Original-Submission.pdf]

Manuscript Click here to download Manuscript bmc_article.pdf

SOFTWARE

Kronos: a workflow assembler for genome analytics and informatics

M Jafar Taghiyar[1,2], Jamie Rosner[1], Diljot Grewal[1,2], Bruno M Grande[3], Radhouane Aniba[1,2], Jasleen Grewal[3], Paul C Boutros[4,5], Ryan D Morin[3], Ali Bashashati *[1,2] and Sohrab P Shah[1,2*]

*Correspondence: sshah@bccrc.ca & abashash@bccrc.ca
[1]Department of Molecular Oncology, British Columbia Cancer Agency, 675 West 10th Ave, V5Z 1L3 Vancouver, BC, Canada
Full list of author information is available at the end of the article

Abstract

Background: The field of next generation sequencing informatics has matured to a point where algorithmic advances in sequence alignment and individual feature detection methods have stabilized. Practical and robust implementation of complex analytical workflows (where such tools are structured into 'best practices' for automated analysis of NGS datasets) still requires significant programming investment and expertise.

Results: We present *Kronos*, a software platform for facilitating ~~automating~~ the development and execution of ~~reproducible,~~ modular, auditable and distributable bioinformatics workflows. Kronos obviates the need for explicit coding of workflows by compiling a text configuration file into executable Python applications. Making analysis modules would still require programming. The framework of each workflow includes a run manager to execute the encoded workflows locally (or on a cluster or cloud), parallelize tasks, and log all runtime events. Resulting workflows are highly modular and configurable by construction, facilitating flexible and extensible meta-applications which can be modified easily through configuration file editing. The workflows are fully encoded for ease of distribution and can be instantiated on external systems, a step towards reproducible research ~~promoting and facilitating reproducible research~~ and comparative analyses. We introduce a framework for building Kronos components which function as shareable, modular nodes in Kronos workflows.

Conclusion: The Kronos platform provides a standard framework for developers to implement custom tools, reuse existing tools, and contribute to the community at large. Kronos is shipped with both Docker and Amazon AWS machine images. It is free, open source and available through PyPI (Python Package Index) and `https://github.com/jtaghiyar/kronos`.

Keywords: genomics; workflow; pipeline; reproducibility

Background

The emergence of next generation sequencing (NGS) technology has created unprecedented opportunities to identify and study the impact of genomic aberrations on genome-wide scales. Data generation technology for NGS is stabilizing and exponential declines in cost have made sequencing accessible to most research and clinical groups. Alongside progress in data generation capacity, a myriad of analytical approaches and software tools have been developed to identify and interpret relevant biological features. These include computational methods for raw data pre-processing, sequence alignment and assembly, variant identification, and variant annotation. However, major challenges are induced by rapid development and

improvement of analytical methods. This makes construction of analytical workflows a near dynamic process, creating a roadblock to seamless implementation of linked processes that navigate from raw input to annotated variants.

~~Most workflow solutions are bespoke, inflexible, and require considerable programming and software development for their implementation.Consequently, the field currently lacks software platforms that facilitate the creation, updating, and distribution of workflows for advanced and reproducible data analysis by clinical and research labs. Robust analysis of large sets of sequencing data therefore remains labor intensive, costly, and requires considerable analytical expertise.~~ As a consequence, robust analysis and continuous iterative improvements in the analysis of large sets of sequencing data remains labor intensive, costly, and requires considerable analytical expertise. As best practices (*e.g.*, [1]) remain a moving target, software systems that can rapidly adapt to new (and optimal) solutions for domain-specific problems are necessary to facilitate high-throughput comparisons.

Several tools and frameworks for NGS data analysis and workflow management have been developed to address these needs. Galaxy [2], is an open, web-based platform to perform, reproduce and share analyses. Using the Galaxy user interface, users can build analysis workflows from a collection of tools available through the Galaxy Tool Shed [3]. The Taverna suite [4] allows the execution of workflows that typically mix web services and local tools. Tight integration with myExperiment [5] gives Taverna access to a network of shared workflows, including NGS data processing.

~~The above tools are mainly aimed at users with minimal programming experience. In addition, Galaxy imposes considerable preparation and installation overhead, lacks explicit representation of workflows (such as in XML format) [6] and imposes some restrictions (such as in file management). Taverna mainly provides a way to run web services and lacks support for scheduling in high performance computing clusters [6].~~

Although the current workflow management systems such as Galaxy are great for routine bioinformatics tasks, development of customized tools and workflows is not convenient and experienced bioinformaticians commonly work at a lower programming level and write their own workflows in scripting languages such as Bash, Perl, or Python [7]. A number of lightweight workflow management tools have been specifically developed to simplify scripting for these target users, including Ruffus [8], Bpipe [9], and Snakemake [10]. Common workflow language (CWL) [11] is a another similar tool that has roots in GNU "make" and aims to build portable workflows across a variety of platforms by using a set of standard specification to define wrappers around command line tools as well as creating nested workflows. While these workflow management tools reduce development overhead, users still need to write a substantial amount of *routine* code to create their own workflows, maintain the existing ones, replace subsets of workflows with new ones, and run subsets of existing workflows.

To further facilitate the process of creating workflows, Omics-Pipe proposed a framework to automate best practice multi-omics data analysis workflows based on Ruffus [12]. It offers several pre-existing workflows and reduces the development overhead for tracking the run of each workflow and logging the progress of each

analysis step. However, it is remains cumbersome to create a custom workflow with Omics-pipe as users need to manually write a Python script for the new workflow by copying/pasting a specific header to the script and writing the analyses functions using Ruffus decorators. The same applies when adding or removing an analysis step to an existing workflow.

We introduce a highly flexible open-source Python-based software tool (Kronos) that enables bioinformatics developers, *i.e.* bioinformaticians who develop workflows for analyzing genomic data, to quickly create a workflow. It uses Ruffus [8] as the underlying workflow management system and adds a level of abstraction on top of it, which significantly reduces programming overhead for workflow development and provides a mechanism to represent a workflow by a top level YAML configuration file.

Kronos is shipped with Docker and Amazon Machine images to further facilitate its use locally, on high performance computing clusters and in the cloud infrastructures. A number of workflows for the analysis of single human genomes and cancer tumour-normal pairs following best analysis practices accompany Kronos and are freely available.

Results

~~Kronos transforms a set of existing *components* (i.e., analysis modules; described later) along with a configuration file into a modular workflow without having to write code. It also provides a functionality to create *component* templates which greatly facilitates developing *components* by experienced bioinformaticians.~~ Kronos creates modular workflows that can be easily updated by editing their corresponding configuration file. Each module in the workflow corresponds to a *component*, which is a wrapped command line tool (i.e., described in more detail later). As shown in Figure 1, users can create a workflow from a set of existing *components* by following three steps listed below (referred to as Steps 1, 2 and 3 in the remainder of this paper). Section 2 of Additional file 1 provides an example of how to make a variant calling workflow.

- Step 1. Given a set of existing *components*, create a configuration file template by running the following Kronos command:

```
kronos make_config
[list of components] -o <output_name>
```

  where [list of components] refers to the *component* names that we aim at using in our workflow.
- Step 2. In the configuration file template, specify the order by which the *components* in the workflow should be run. This does not require programming skills and is merely text-based.
- Step 3. Create the workflow by running the following Kronos command with the configuration file as its input:

```
kronos init -y <config_file.yaml>
-o <workflow_name>
```

The output is an executable Python script that uses the built-in run manager of Kronos. The run manager provides scalability by enabling users to run the workflow on a single machine, on a cluster of computing nodes or in the cloud . In fact, each *component* in the workflow can individually be run either locally, on a cluster. In addition, it allows users to independently set native specifications such as free memory, maximum memory or the number of CPU's, for each task. The run manager also provides the following features for the resulting workflow:

- generates a unique run-ID for each run
- re-runs the workflow from where it left off using the run-ID
- runs intermediate workflows in parallel
- limits the number of concurrent jobs and workflows as desired
- creates a detailed log file for each run tagged with the run-ID

The output is an executable Python script that runs the workflow. Depending on its corresponding configuration file, the script is encoded to automatically parallelize eligible tasks, provide pause/resume functionality, make unique run ID's, make the desired output directory tree, submit jobs to cluster or run them locally, and log the events.

Kronos *components*

In order for different software tools, referred to as *seeds*, to be used as input to the `make_config` command (Step 1), users need to wrap them with a number of particular files. We call a wrapped *seed* a *component*. A *seed* can be as simple as a command copying a file or it can be a more complicated tool such as a single nucleotide variant (SNV) caller.

Regardless of how complicated a *seed* is, its corresponding *component* has a standard directory structure composed of specific wrappers and sub-directories. The wrappers are independent of the programming language used for developing the *seed* and essentially all tools can be wrapped as *components*. In addition, Kronos provides a functionality (through `make_component` command) to create a *component* template which helps developing a new *component* in a few minutes (Figure 2) provided that the *seed* exists. This process is straight-forward and requires minimal programming, yet it provides a powerful framework for experienced programmers to fully customize their workflows. Section 1 of Additional file 1 provides an example creating a *component*.

A *component* is a wrapper around a command line tool that encapsulates all the required programming. The purpose of *components* is to modularize workflows with reusable building blocks that require minimal development. As shown in Section 1 of Additional file 1, the number of lines of codes for making a new *component* is very small. The simple development instructions eliminate, for example, the need to use Ruffus decorators, input/output management using regex expressions and complicated dependency management in the code that can easily become very complex with the number of tasks in a workflow. Furthermore, a large workflow can be divided into a set of small *components* that results in a much faster and manageable workflow development. Kronos also provides a command for making *component* templates that helps developing a new component in a few minutes.

All command line tools, such as a simple copy command or a complicated single nucleotide variant (SNV) caller, can be wrapped as Kronos *components*. Regardless

of how complicated they are, their corresponding *components* have a standard directory structure composed of specific wrappers and sub-directories. The wrappers are also independent of the programming language used for developing the command line tool.

The *components* should be developed prior to making the workflow. However, since they are individually and independently developed and due to their reusability, the initial preparation of a *component* happens only once and various workflows can use the already developed *component*.

Kronos configuration file

Kronos workflows are represented by a YAML configuration file. For a given set of *components*, Kronos `make_config` command generates a configuration file template that is mostly pre-filled with default values. For each input *component*, there is a corresponding section with a unique name in the configuration file called *task*. Users should use these sections to specify the order by which each *task* in the workflow should be run (Step 2 of creating a workflow). This can be done by a simple convention called *IO-connection*. An *IO-connection* is basically a pair of values comprising of a *task* name and one of its parameters. It determines which *task* should be followed by the current *task* and is specified as an argument to one of the parameters of the current *task*. For example, in the following configuration file, `('__TASK_1__', 'out_file')` is an *IO-connection* which makes `__TASK_2__` to follow `__TASK_1__`, *i.e.* the input to the parameter `in_file` of `__TASK_2__` comes from the parameter `out_file` of `__TASK_1__`.

```
__TASK_1__:
    out_file: 'foo.txt'
__TASK_2__:
    in_file: ('__TASK_1__', 'out_file')
```

The run options for each task are also set in the configuration file including granular resource requests such as free memory or the number of CPU's, running locally or on cluster, running with parallelization, pause/resume functionality, etc.

A configuration file has the following blocks (see Additional file 1: Figure S1):

- system-specific which captures the system dependant requirements of the workflow (such as the paths to the local installations) and includes the `GENERAL` and `PIPELINE_INFO` sections.
- user-specific which contains the input files and arguments and includes the `SHARED` and `SAMPLES` sections.
- workflow-specific which defines the connection between the *components* in the workflow. *Task* sections related to each *component* are in this group.

This design has the following advantages: i) if users want to re-run the same workflow for various sets of input files and arguments, they would only need to update the user-specific sections. This prevents inadvertent changes in the flow of the workflow when changing the inputs; and ii) the segregation of system-specific information from the rest of the sections enables users to run a workflow practically anywhere. In other words, by simply updating the system-specific sections with proper values, the requirements of the workflow can be observed on any machine.

Kronos workflows

Each workflow made by Kronos is a directed acyclic graph (DAG) of *components* where every node in the graph corresponds to a *task* section in the configuration file. *Task* sections can independently be added, removed or replaced in the configuration file (Figure 3). Therefore, to add, remove or replace a *component* in the workflow or equivalently a node in the DAG, users simply need to change the corresponding *task* section in the configuration file and run the command in Step 3. As a result, the workflows are highly modular and maintaining them is as easy as updating the configuration file without having to rewrite the workflow. Finally, a workflow can be run by simply running the Python workflow script using the command-line as depicted in Figure 4.

Kronos features and benefits

Full details of how to use each of the following features can be found in the software documentation.

Parameter sweeping

It is sometimes desired to run a particular tool or algorithm with various sets of parameters in order to select the parameter set with superior performance for a given problem. For example, a user may want to find the proper model parameters (such as mapping quality and base quality thresholds) for a variant calling tool to accurately detect single nucleotide variants. Kronos provides a mechanism for this purpose where users can specify all different sets of input arguments (or parameters) in the `SAMPLES` section of the configuration file. In this case, running Step 3 creates a number of intermediate workflows, each for one set of input arguments, along with the main workflow. When running the main workflow, Kronos runs the intermediate workflows in parallel, each on one set of the input arguments. We have provided a variant calling workflow with parameter sweeping functionality in Section 3 of Additional file 1 to demonstrate this feature.

Tool comparison

In bioinformatics, it is often required to compare the performance of two or more algorithms or compare a new analysis tool to the existing ones to select the best that fits the particular goals of a project. For example, it is often helpful to evaluate the performance of different variant calling algorithms [13]. The modularity of the workflows generated by Kronos facilitates the comparison of different algorithms and tools. For this purpose, as shown in Figure 3, the user can simply replace a *task* section corresponding to an analysis tool with another *task* section corresponding to another similar tool and run Step 3.

Automatic parallelization and merge

Most of the recent tools developed in bioinformatics field are parallelizable or have the potential to run in parallel. However, majority of these tools are shipped without the built-in functionality and require the users to manually break the analysis into smaller analyses. For example, many variant calling algorithms are capable of running on user-specified coordinates of the genome but are not shipped with parallelization functionality. However, a user can analyze a whole genome sequencing

data chunk by chunk in parallel with the caveat of manually scripting the parallelization steps. Due to the cumbersome nature of manual parallelization, many users might avoid running the tools in parallel which considerably increases the runtime of the analysis. To resolve this issue, Kronos automatically parallelizes tasks in the workflow if feasible. Then, it aggregates the outputs of all child tasks and merges them if necessary.

~~Reproducibility~~ Reproducible workflows

The configuration file and *components* of a workflow are portable. ~~Therefore, users can readily re-create the same workflow by only replacing the system-specific section of the configuration file and running the command in Step 3.~~ Therefore, users can readily duplicate a workflow elsewhere by only running the `kronos init` command in Step 3. To show this functionality, we have included an example of a workflow that performs somatic variant calling on whole genome data of a breast cancer case using Strelka algorithm [14] and generates a number of plots based on Strelka calls (Figure 5). Detailed step-by-step instructions to reproduce this figure is in Section 3 of Additional file 1. It should be noted that Kronos workflows can be duplicated elsewhere but the user would still need to manage tool installations and dependencies.

Cloud support

The massive scale of genomic data is justifying a move to the cloud for storage and analyses in order to minimize cost and handle the ebb and flow of computational demands. Kronos' flexibility addresses the emerging need for rapid deployment of analysis workflows in the cloud. Several command-line tools exist for managing fleets of compute nodes on cloud platforms such as Amazon Web Services (AWS), including StarCluster, CfnCluster and Elasticluster. ~~Kronos is scheduler-agnostic; therefore, developers can leverage its powerful features in combination with any of these tools.~~ A guide on creation and management of a cloud cluster using the StarCluster software and deployment of Kronos is provided in the online documentation and an Amazon Machine Image (AMI) is provided for convenience.

Controlled pause/resume by breakpoints

When running a workflow, certain blocks of the workflow may need to run multiple times, for example to tune a particular parameter of a *component* or to inspect the results of the previous *tasks* in the workflow before the next *tasks* are triggered. Analogous to the debuggers, Kronos provides users with breakpoints to perform a controlled pause/resume action.

In addition, with the breakpoint mechanism, users can break the flow of a workflow into several sub-workflows and run each part on a different machine or cluster. In other words, once a breakpoint happens, *i.e.* one sub-workflow is complete, the main workflow can be transferred to a different machine and it will pick up running from where it left off on the previous machine provided that all the intermediate files are present. For example, a workflow can contain a *component* as its last step that loads the final results to a local database which can be reached only from a specific IP or machine. In this case, the user can run the workflow on a powerful computing node

or a cluster with a breakpoint set for the *component* prior to the last *component*, *i.e.* database loader in this example. Once the breakpoint is applied, the user can resume the workflow on the other machine, so that the results can be loaded to the local database.

Forced dependency
Often in a workflow, a *task* requires the output of the previous one. As explained earlier, Kronos handles this explicit dependency by *IO-connection*. However, sometimes a *task* might need to wait for one or more other steps in the workflow to finish although there are no explicit *IO-connections* between them. For example, when two tasks intend to write results in the same file, one needs to make sure that both tasks do not run at the same time. Another example would be a variant calling algorithm (*e.g.*, GATK) which accepts a bam file as input. However, it also expects the index of the bam file to be present in the same directory as the bam file. If the index is created in one of the previous *tasks* in the workflow, then the current *task* that needs the bam file and its index, would depend implicitly on the other *task* that creates the index file. In this case, a mechanism is required to force the variant calling *task* to wait until the index file is ready. Kronos provides forced dependency feature to overcome this problem (see Additional file 1: Figure S2).

Results directory customization
It is desirable to have full control of the structure of the results directory when running a workflow. With Kronos, users can readily determine the structure of the results directory in the configuration file. This provides an easy file management for the users. Figure 4 shows an example of the tree structure of the results directory generated for a workflow.

Boilerplates
Users can use this feature to insert a command or a script into the begining of the command used to run a *task* in a workflow. This is particularly useful for setting up the environments using the Environment Modules package [15]. It also provides a means to run preprocessing steps for a specific *task* prior to running the *task* itself.

Keywords
There are several specific keywords that users can use in the configuration file which will be automatically replaced by proper values in runtime. This enables users to customize the paths and file names based on the workflow-specific values in runtime such as run-ID, workflow name or sample ID.

Workflows
We have developed a number of standard genome analysis workflows using Kronos. These workflows utilize many of the Kronos features introduced earlier and are publicly available.

Alignment workflow This workflow accepts paired-end FASTQ files as input and aligns them using the Burrows-Wheeler aligner [16]. It also sorts the aligned bam file, flags the duplicates, indexes the file and generates statistics for the final bam file.

Germline variant calling workflow This workflow is an implementation of the best practices guide established by the Broad Institute [1] applied to variant discovery using haplotypecaller. In short, it runs the Bowtie2 aligner, creates targets using GATK RealignerTargetCreator, and calls SNVs and indels using GATK.

Copy number estimation workflow HMMcopy is a suite of tools for copy number estimation of whole genome sequencing data [17]. This workflow takes a bam file as an input and estimates the copy number with GC and mappability correction using HMMCopy. It also segments and classifies the copy number profiles with a robust Hidden Markov Model.

Somatic variant calling workflow This workflow takes a pair of tumour/normal bam files as inputs and detects the somatic SNVs and indels using Strelka algorithm [14], annotates the resulting VCF files using SnpEff [18], and flags the variants observed in 1000 genomes and dbSNP databases.

RNA-seq analysis workflow This workflow aligns RNA-seq FASTQ files using STAR aligner [19] followed by Cufflinks which assembles transcriptomes from RNA-Seq data and quantifies their expression [20].

Conclusions

A foundation for rapid and reliable implementation of genomic analysis workflows is an essential need as a myriad of potential applications of genomics (ranging from personalized cancer therapies to monitoring the evolution and spread of infectious diseases) are projected to produce massive amount of genomic data in the next few years. ~~We have developed Kronos to address this need and standardize reproducibility of genome analysis tasks. Kronos minimizes the cumbersome process of writing code for a workflow by transforming a YAML configuration file into a Python script and manages workflow execution locally, on a cluster or in the cloud.~~ We have developed Kronos to address this need by expediting workflow development. It minimizes the tedious process of writing code by transforming a YAML configuration file into a Python script and manages its execution. Given a set of pre-made *components*, constructing a workflow by Kronos does not need programming skills as the user only needs to fill out specific sections of the configuration file. Making *components* still requires programming. ~~However, component development time and effort is minimal given the design structure of Kronos' *components*. In addition, Kronos provides a functionality to create a *component* template that can be used to wrap an existing software (*seed*) with minimal coding intervention. This provides a powerful and highly flexible framework for bioinformaticians to fully customize their workflows.~~ However, their development time and effort is minimal given their design structure. They also provide a powerful and highly flexible framework for bioinformatics developers to fully customize their workflows with reusable modules.

A number of standard genomic analysis workflows along with their building *components* that have been made by Kronos accompany this software and are available to public. Kronos has been developed for genomics applications but it can be readily utilized in other scientific and non-scientific fields.

The configuration file and *components* of a Kronos workflow are portable. This is a step towards reproducible research; however it should be noted that while Kronos workflows can be duplicated elsewhere, the user would still need to manage tool installations and dependencies. For a full reproducible research, a Docker image of the whole workflow or the environment is perhaps more plausible. Kronos is complementary to other efforts for reproducible research. For example, in order to unify representation of workflow definitions and tool wrappers, the Common Workflow Language (CWL) working group [11] and the Workfow Description Language (WDL) [21] offer specifications that enable data scientists to describe analysis tools and workflows that are human readable, easy to use, portable, and support reproducibility. It would be beneficial for workflow management tools to adopt these representation standards once they are agreed upon in the field.

In conclusion, this work provides a framework towards rapid integration of new (and optimal) genomic analysis advances in high-throughput studies. The flexibility, customization, and modularity of Kronos make it an attractive system to use in any high-throughput genomics analysis endeavour. We expect Kronos will provide a foundational platform to accelerate towards the need to standardize and distribute NGS workflows in both clinical and research applications.

Availability and requirements

Kronos is a free and open-source Python package available through PyPI (Python Package Index) under the MIT license. Documentation can be found at `https://readthedocs.org/projects/kronos/` and the workflows and their *components* accompanying this paper are available at `https://github.com/MO-BCCRC?tab=repositories`.

Competing interests

The authors declare that they have no competing interests.

Author's contributions

J.T. developed the software, wrote the documentation and contributed to manuscript writing. J.R. assisted in developing part of the logger and a few of the helper functions for the software, testing software features and providing feedback on the manuscript. D.G. developed a number of pipelines accompanying the manuscript, tested the software and provided feedback on the software features and manuscript. B.G. deployed and tested Kronos in the cloud, wrote the documentation for cloud deployment, tested software and provided feedback on the software features and manuscript. R.A. developed the germline variant calling workflow and provided feedback on the manuscript. J.G. tested and provided feedback on the software. P.B. provided feedback on the manuscript. R.M. provided resources and supervised testing Kronos in the cloud, and provided feedback on the manuscript. A.B. contributed to the design and development of the software. A.B. and S.S. co-supervised, provided intellectual contributions to the work and contributed to manuscript writing. A.B. and S.S. are joint senior authors.

Acknowledgements

The authors would like to thank Shadrielle Melijah G. Espiritu and Andre Masella for their feedback on the manuscript/software. This project has been supported by funding from Genome Canada/Genome British Columbia (grant No. 173CIC), Natural Science & Engineering Research Council of Canada (grant No. RGPGR 488167-2013), and Terry Fox Research Institute - Program Project Grants (grant No. 1021).

Author details

[1]Department of Molecular Oncology, British Columbia Cancer Agency, 675 West 10th Ave, V5Z 1L3 Vancouver, BC, Canada. [2]Department of Pathology and Laboratory Medicine, University of British Columbia, 2211 Wesbrook Mall, V6T 2B5 Vancouver, BC, Canada. [3]Department of Molecular Biology and Biochemistry, Simon Fraser University, 8888 University Drive, V5A 1S6 Burnaby, BC, Canada. [4]Ontario Institute for Cancer Research (OICR), 661 University Avenue, M5G 0A3 Toronto, ON, Canada. [5]Department of Medical Biophysics, University of Toronto, 101 College Street, M5G 1L7 Toronto, ON, Canada.

References

1. GATK Best Practices - Recommended Workflows for Variant Analysis with GATK. `https://www.broadinstitute.org/gatk/guide/best-practices`
2. Goecks, J., Nekrutenko, A., Taylor, J., Galaxy Team, T.: Galaxy: a comphrehensible approach for supporting accessible, reproducible, and transparent computational research in the life sciences. Genome biology **11(R86)**, 1–13 (2010)
3. Galaxy Tool Shed. `https://toolshed.g2.bx.psu.edu`
4. Wolstencroft, K., Haines, R., Fellows, D., Williams, A., Withers, D., Owen, S., Soiland-Reyes, S., Dunlop, I., Nenadic, A., Fisher, P., Bhagat, J., Belhajjame, K., Bacall, F., Hardisty, A., Nieva de la Hidalga, A., Balcazar Vargas, M., Sufi, S., Goble, C.: The taverna workflow suite: designing and executing workflows of web services on the desktop, web or in the cloud. Nucleic acids research **41**, 557–561 (2013)
5. Goble, C.A., Bhagat, J., Aleksejevs, S., Cruickshank, D., Michaelides, D., Newman, D., Borkum, M., Bechhofer, S., Roos, M., Li, P., De Roure, D.: myexperiment: a repository and social network for the sharing of bioinformatics workflows. Nucl. Acids Res **38(2)**, 677–682 (2010)
6. Abouelhoda, M., Alaa, S., Ghanem, M.: Meta-workflows: Pattern-based interoperability between galaxy and taverna. In: Proceedings of the 1st International Workshop on Workflow Approaches to New Data-centric Science. Wands '10, pp. 2–128. ACM, New York, NY, USA (2010). doi:10.1145/1833398.1833400. http://doi.acm.org/10.1145/1833398.1833400
7. Spjuth, O., Bongcam-Rudloff, E., Hernández, G.C., Forer, L., Giovacchini, M., Guimera, R.V., Kallio, A., Korpelainen, E., Kańduła, M.M., Krachunov, M., *et al.*: Experiences with workflows for automating data-intensive bioinformatics. Biology direct **10**(1), 1–12 (2015)
8. Goodstadt, L.: Ruffus: A lightweight python library for computational pipelines. Bioinformatics **26(21)**, 2778–2779 (2010)
9. Sadedin, S., Pope, B., Oshlack, A.: Bpipe: A tool for running and managing bioinformatics pipelines. Bioinformatics **28(11)**, 1525–1526 (2012)
10. Koster, J., Rahmann, S.: Snakemake - a scalable bioinformatics workflow engine. Bioinformatics **28(19)**, 2520–2522 (2012)
11. Common Workflow Language (CWL). `http://www.commonwl.org/draft-3/index.html`
12. Fisch, K.M., Meißner, T., Gioia, L., Ducom, J., Carland, T.M., Loguercio, S., Su, A.I.: Omics pipe: a community-based framework for reproducible multi-omics data analysis. Bioinformatics (2015)
13. Ewing, A.D., Houlahan, K.E., Hu, Y., Ellrott, K., Caloian, C., Yamaguchi, T.N., Bare, J.C., P'ng, C., Waggott, D., Sabelnykova, V.Y., et al.: Combining tumor genome simulation with crowdsourcing to benchmark somatic single-nucleotide-variant detection. Nature methods (2015)
14. Saunders, C.T., Wong, W.S., Swamy, S., Becq, J., Murray, L.J., Cheetham, R.K.: Strelka: accurate somatic small-variant calling from sequenced tumor–normal sample pairs. Bioinformatics **28**(14), 1811–1817 (2012)
15. Environment Modules Project - Software Environment Management. `http://modules.sourceforge.net/`
16. Li, H., Durbin, R.: Fast and accurate short read alignment with burrows-wheeler transform. Bioinformatics **25**, 1754–60 (2009)
17. Ha, G., et al.: Integrative analysis of genome-wide loss of heterozygosity and mono-allelic expression at nucleotide resolution reveals disrupted pathways in triple negative breast cancer. Genome Research **22(10)**, 1995–2007 (2012)
18. Cingolani, P., Platts, A., le Wang, L., Coon, M., Nguyen, T., Wang, L., et al.: A program for annotating and predicting the effects of single nucleotide polymorphisms, snpeff: Snps in the genome of drosophila melanogaster strain w1118; iso-2; iso-3. Fly **6**, 80–92 (2012)
19. Dobin, A., Davis, C.A., Schlesinger, F., Drenkow, J., Zaleski, C., Jha, S., Batut, P., Chaisson, M., Gingeras, T.R.: Star: ultrafast universal rna-seq aligner. Bioinformatics **29**(1), 15–21 (2013)
20. Trapnell, C., Roberts, A., Goff, L., Pertea, G., Kim, D., Kelley, D.R., Pimentel, H., Salzberg, S.L., Rinn, J.L., Pachter, L.: Differential gene and transcript expression analysis of rna-seq experiments with tophat and cufflinks. Nature protocols **7**(3), 562–578 (2012)
21. Workflow Description Language (WDL). `https://github.com/broadinstitute/wdl`

Figures

Figure 1 Make a workflow. Making a new workflow with Kronos includes three steps: i) make a configuration file template: given a set of existing *components*, users can generate this file by running the command `make_config`; ii) configure the workflow: users can specify the desirable flow of their workflow using the connections and dependencies, customize output directory names, and specify input arguments and data to the required fields in the configuration file template; iii) initialize the workflow: this is achieved by running the command `init` on the configuration file which transforms the YAML file into the Python workflow script.

Figure 2 Make a component. Making a new *component* for Kronos includes the following steps: i) make a new *component* template by running the command `make_component`; ii) fill in the resulting template accordingly; iii) copy or link the source code of the *seed* used in the *component*; iv) optionally create `README.md` and tests for the *component*.

Figure 3 Replace a component in a workflow. The configuration file has different sections as shown in the figure. These sections are: GENERAL, PIPELINE_INFO, SHARED, SAMPLES, and TASKs. The modular organization of the configuration file allows for easy customization of workflows which can serve different purposes such as tool comparison. Adding, removing or replacing nodes in the DAG of the workflows can be easily done by adding, removing or replacing the corresponding TASK sections in the configuration file. For instance, to go from workflow `DAG1` to workflow `DAG2`, *i.e.* to replace `comp_1` (*e.g.*, variant caller 1) in the first workflow by `comp_5` (*e.g.*, variant caller 2) in the second, the user only needs to replace `TASK_1` section by `TASK_5` section in the configuration file and perform Step 3.

Figure 4 Run a workflow. Workflows generated by Kronos are ready to run locally, on a cluster of computing nodes, and in the cloud. To run a workflow, users only need to run the Python workflow script. Each run of a workflow generates a specific directory structure tagged with a run-ID. When running a workflow for multiple samples, a separate directory is made for each sample to make it convenient to locate the results corresponding to each sample. This figure shows the tree structure of the resulting directory. There are four sub-directories that are always generated for each sample: i) logs: to store the log files; ii) outputs: to store all the output files generated by all the *components* in the workflow ; iii) scripts: to store the scripts automatically generated by Kronos to run each *component* in the workflow ; iv) sentinels: to store sentinel files used by Kronos to pick up the workflow from where it left off in a previous run.

Figure 5 Strelka workflow. Results from the tumour-normal variant calling workflow on whole genome data of a breast cancer case (SA500 - EGA accession number EGAS00001000952). (A) schematic of the workflow which is comprised of two tasks. The plots generated by the workflow is in fact the output of TASK_2, (B) box plot of coverage and variant allelic ratios for the SNVs detected by Strelka, (C) base substitution patterns for the somatic SNVs, and (D) total number of SNVs and their histogram based on the quality score (QSS), (E) Distribution of the number of SNVs across different chromosomes.

Additional Files

Additional file 1 — Supplementary information

The supplementary information is in pdf format and expalins a) how to make a *component*, b) how to make a workflow, c) how to run a workflow, and d) Figure S1 and Figure S2.

Figure 1

Click here to download Figure
kronos_make_new_workflow.pdf

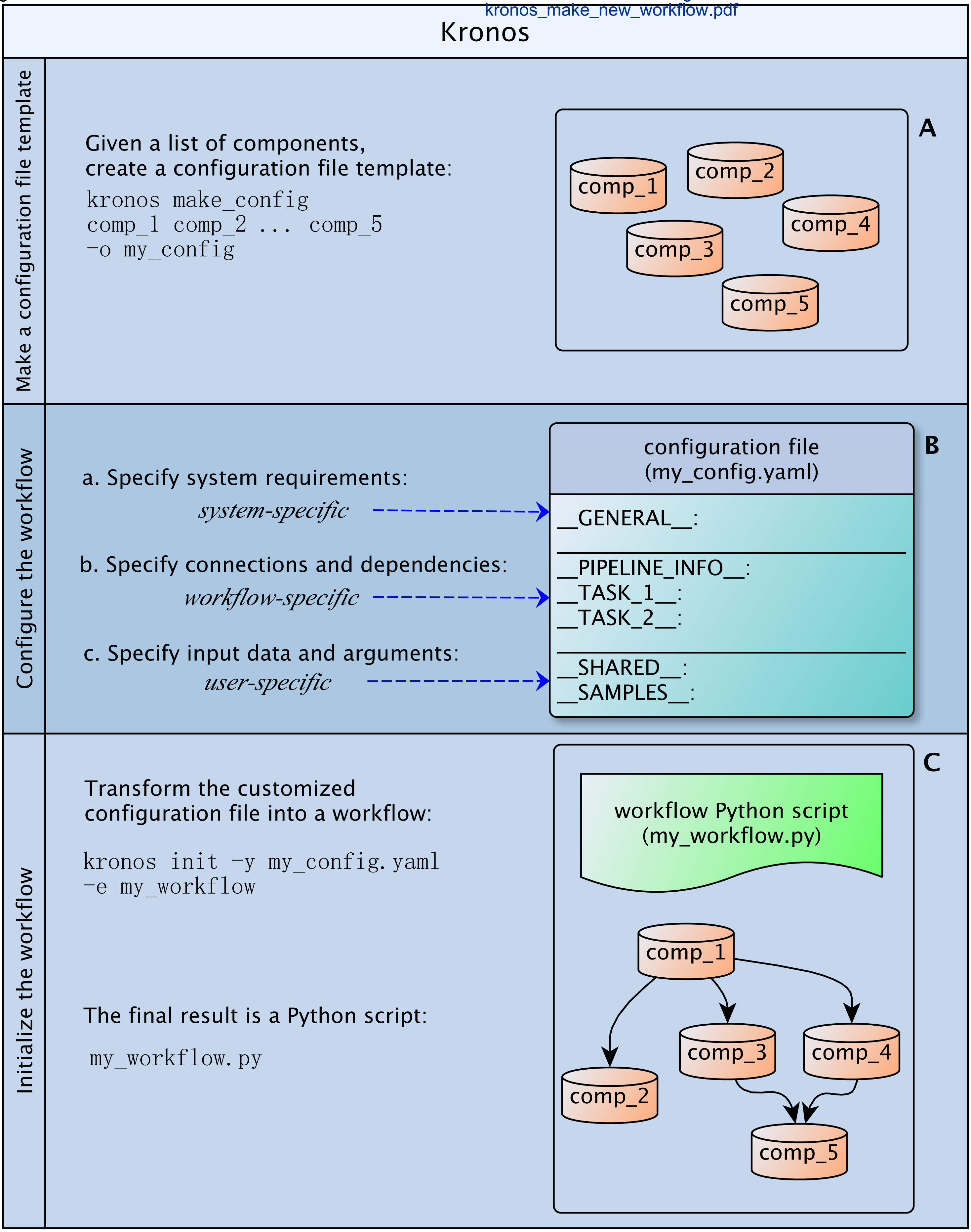

Figure 2 Click here to download Figure kronos_make_new_component.pdf

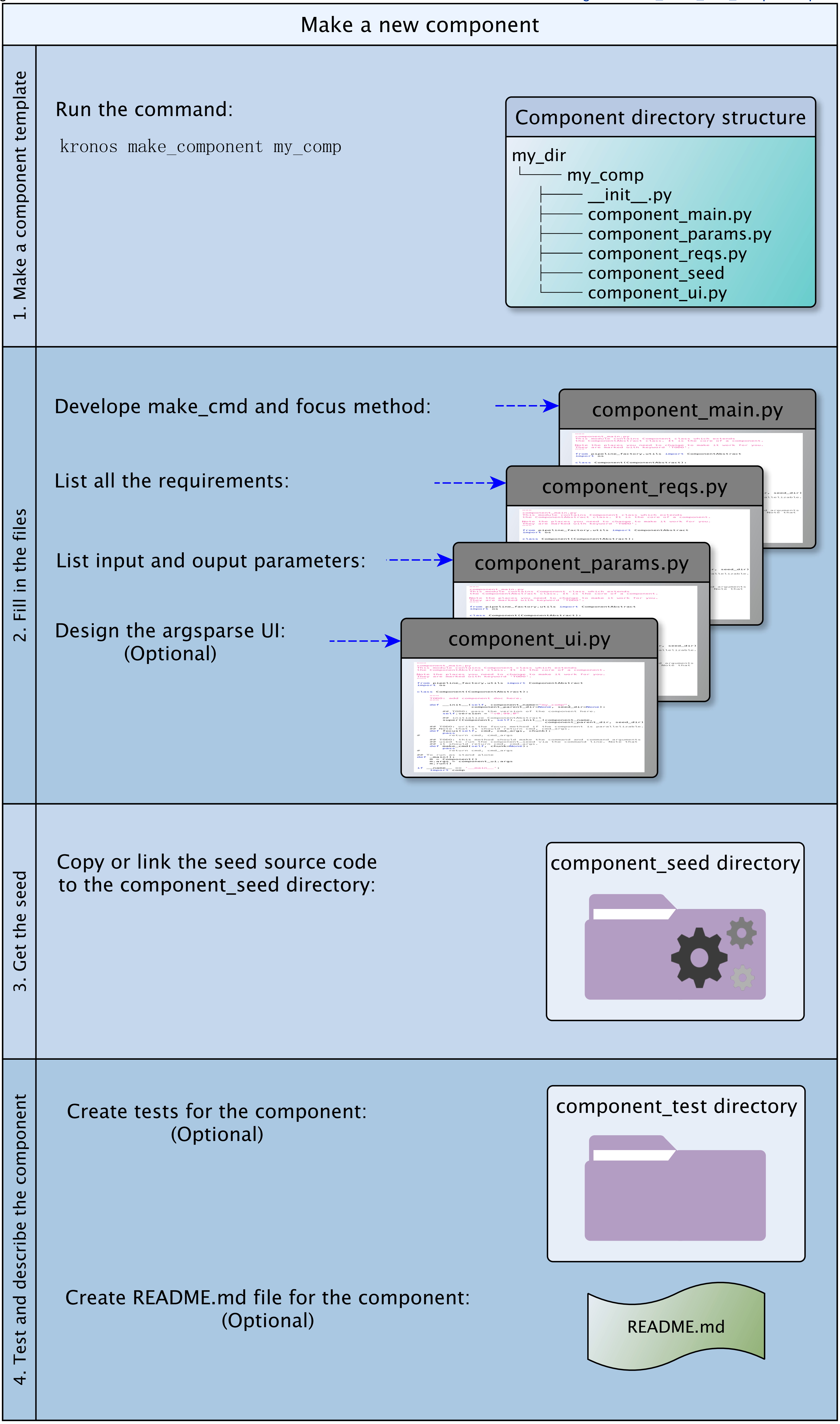

Figure 3

Click here to download Figure kronos_modular_workflow.pdf

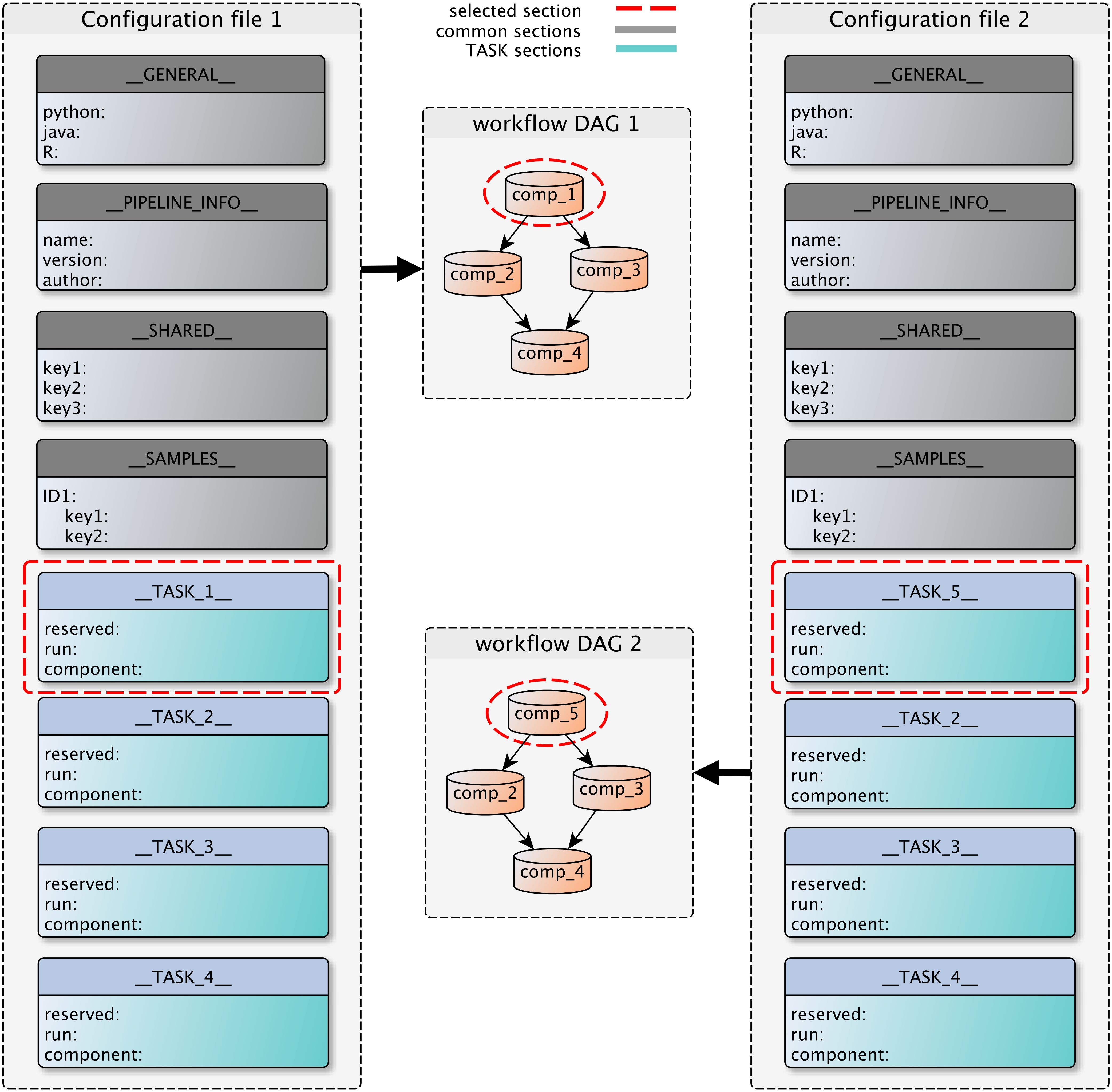

Figure 4

Click here to download Figure kronos_run_workflow.pdf

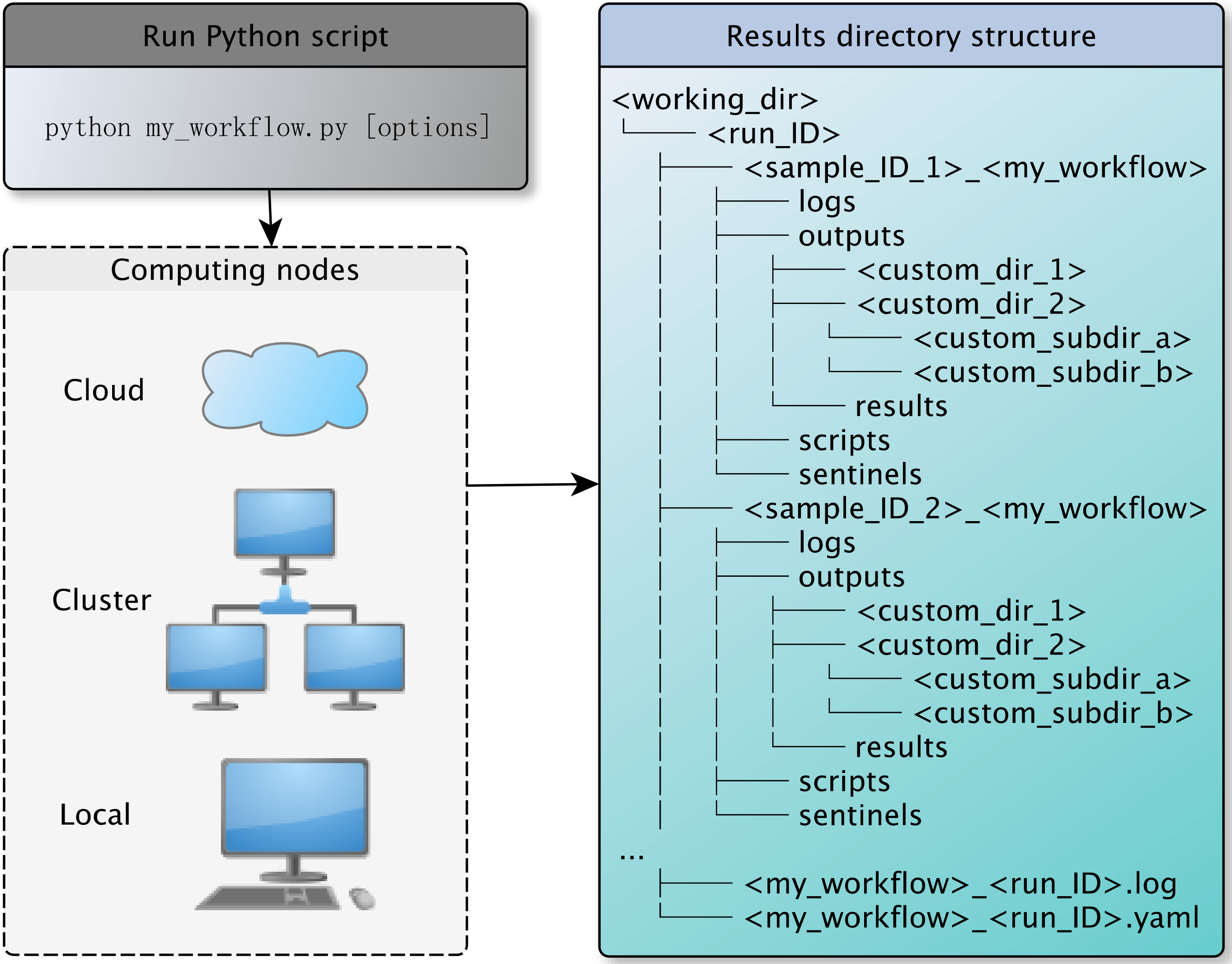

Figure 5

Click here to download Figure strelka_portrait_SA500.pdf

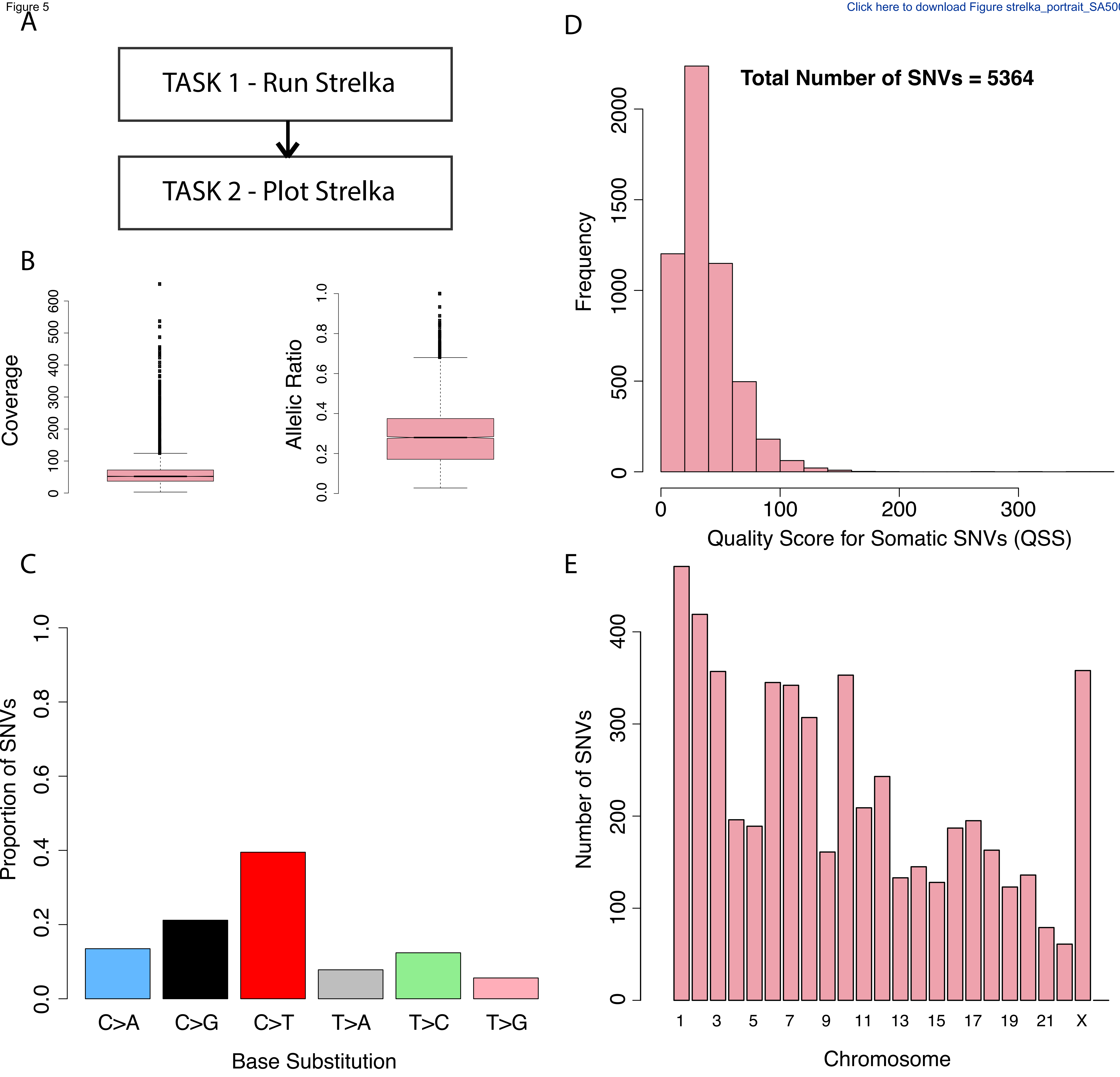

Click here to access/download
Supplementary Material
supplementary_info.pdf

Cover letter including the response letter
Click here to download Personal Cover response_letter.docx

Feb 1, 2017

Dr. Scott Edmunds, Executive Editor, GigaScience

Re: GIGA-D-16-00013 - Kronos: a workflow assembler for genome analytics and informatics

Dear Dr. Edmunds,

Thank you for sharing the helpful and constructive reviews of our submission. We have now improved the manuscript with all comments addressed. In particular, as requested, we have clarified the presentation regarding the utility of our software, where it fits in the workflow ecosystem, and the value that it adds to Ruffus.

Below you will find a point-by-point response with all comments from the two reviewers addressed. We have also highlighted the changes in the main text.

I look forward to receiving the reviews of our revised manuscript and your editorial decision in due course.

With best wishes,

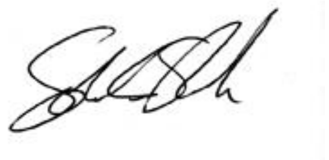

Dr. Sohrab Shah, Ph.D.
Canada Research Chair in Computational Cancer Genomics
University of British Columbia
British Columbia Cancer Agency
Email: sshah@bccrc.ca
Phone: +1 604 675 8252

Reviewer #1 (Bjoern Groening):

The manuscript entitled "Kronos: a workflow assembler for genome analytics and informatics" by Taghiyar and colleagues introduces and describes a software platform for automating the development and execution of workflows in bioinformatics.

Despite the very well written and detailed paper I'm missing the novelty of the described platform.

Response: Kronos is not a new workflow management system. It uses Ruffus as the underlying workflow management system and adds a level of abstraction on top of it to significantly reduce the programming overhead for workflow development. This is the main novelty and motivation behind developing this platform. Furthermore, Kronos adds several important features on top of Ruffus (see “Kronos Features and Benefits” Section) as well as provides a mechanism to represent a workflow by a top level YAML configuration file. We have updated the text to clarify this (please see page 3 second paragraph and “Kronos configuration file” subsection).

The comparison with other workflow management systems like Taverna, Galaxy and Snakemake is insufficient and, to my knowledge, wrong in certain instances.
A few remarks to this comparison:
* Taverna & Galaxy are aimed at users without programming experience
This was maybe true at the beginning, but today Galaxy offers capabilities for experienced users. Those users can use the API to script Galaxy and execute workflows, retrieve results ...
In addition with the integration of IPython or the more recent Jupyter Notebook it is even possible to program in 5 different languages in the Galaxy web-UI, which makes it idea suited for people that want to combine workflows with ad-hoc scripting.
* Galaxy is hard to install
A user doesn't need to install Galaxy, as he can use one of the public available Galaxy Servers out there. If you want to have your own private Galaxy instance the community provides you with Galaxy Docker containers (production ready), ansible playbooks and VMs.
* lacks explicit representations of workflows
In Galaxy workflows can be represented as YAML or JSON file, which I consider an explicit representation.
Speaking about workflows, initiatives like CWL, WDL are not discussed in this context. Even not strictly needed in such a manuscript, if the reason to create a new workflow platform is an explicit representation I would expect a discussion about this and how Kronos is better than other solutions.
Response: Agreed. We had initially referred to a comparison that was presented in Abouelhoda et al. 2010. We have now revised the text to address this issue and better reflect the recent changes in Galaxy (please see “Background” section).
Abouelhoda, Mohamed, Alaa, Shady and Ghanem, Moustafa, Meta-workflows: Pattern-based Interoperability Between Galaxy and Taverna, Proceedings of the 1st International Workshop on Workflow Approaches to New Data-centric Science, 2010, Indianapolis, Indiana,
pp. 2:1-2:8.

* restrictions such as in file management
This was not entirely clear to me.
Response: From our experience working with Galaxy, files can either be uploaded (via HTTP or FTP) or externally linked when using Galaxy. When uploaded, a copy of the file gets created on the Galaxy server. If Galaxy is running on a server that has access to the input files, users can externally link to these files to avoid creating duplicates. This being said, regardless of which method is used to add input files to Galaxy, all intermediate and output files are created and managed internally by Galaxy.

Power users are less able to access these files directly without going through the interface, which is rate-limiting when trying to access hundreds of files, not to mention that files obtained from the interface are downloaded to the computer accessing the web interface. This isn't practical when Galaxy is running on a server where all your files need to be located due to their large size. Unfortunately, the intermediate and output files have opaque names on disk, such as “dataset_79.dat”, preventing power users from accessing them directly.

This also poses an issue when managing access permissions to restricted data for specific users. While the web interface allows the creation of user accounts, files on disk would have to be completely inaccessible to all users in order to prevent unauthorized access, thereby worsening the problem of accessing files on disk for power users.

* Ruffus, Bpipe or Snakemake
The authors state that the advantage of Kronos is that here a developer does not need to write a substantial amount of code and that copy and pasting is required to create workflows. This seems to me very subjective or individual perspective and I'm wondering how this justifies a new workflow managment system that is targeted at developers that like to program.
Response: As mentioned earlier, Kronos is not a new workflow management system. It mainly aims at facilitating workflow development for bioinformatics developers. These developers need to frequently make, update and maintain various workflows. They also mostly use lightweight workflow tools such as GNU make, Snakemake, Ruffus, etc. We have chosen Ruffus which is a very powerful workflow management system. It requires handling complex regex expressions, decorating functions, dependency management, etc that introduce cumbersome programming overhead. However, these steps can be automated and Kronos saves bioinformatics developers considerable time by automating them following the “convention over configuration” philosophy. This helps to make the routine work of development a workflow using Ruffus much faster while keeping its flexibility.
We have now changed the text to clarify these issues (please see page 3 second paragraph).

Moreover, looking at the example code of some 'components' I don't see a big difference in writing ruffus instructions or Kronos components.
Response: The difference between Kronos components and Ruffus instructions is that Kronos automatically handles the programming overhead of Ruffus. Therefore, Kronos components are very simple wrappers that at most need handful lines of code. For larger workflows, not only this is a significant advantage that makes it possible to create a workflow in a fraction of time needed otherwise using Ruffus, but also makes the components very succinct and independent of the complexity of the workflow. Kronos also provides a command to make a component template that enables users to create a new component quickly.
We believe that the text was not clear enough to show the differences. We have now fixed this issue (please see “Kronos components” subsection).

Technical remark:
* creating indices of all kind should be a core feature and not requiring an additional task
Response: This was only an example to show the use case of the “forced_dependency” feature. In the mentioned example, the GATK task requires both a bam file and its index as inputs. However, only the bam file is generated by the aligner task and not the index of the resulting bam file (this is not the core feature of any aligners to make the index files simultaneously to our best knowledge). Therefore, another task was needed to make the corresponding index file.

In general, different index files for different tools often require their own mechanism. For example, STAR aligner [1,2] has a custom built-in functionality for generating its required genome indices for the reference FASTA file. From its user manual [3]:
“Genome files comprise binary genome sequence, suffix arrays, text chromosome names/lengths, splice junctions coordinates, and transcripts/genes information. Most of these files use internal STAR format and are not intended to be utilized by the end user. It is strongly not recommended to change any of these files.”

As a result, we do not believe that creating all types of indices should be a core feature of Kronos. In addition, making an index is a simple task and can be easily wrapped to a Kronos component and called in the workflow when necessary.

Essentially, “forced_dependency” feature is useful whenever in a workflow a task needs to wait for another task before execution while there are no explicit dependencies between the two.

Another example is a cleanup task. It needs to wait for other tasks to finish while it has no direct connections to them via inputs/outputs.

* Is there a concept of filetypes and some checking if a component can work with some specific filetypes?
Response: Currently, this is done as part of the component. In other words, if a component works only with a specific filetype, then the “component_main.py” checks if the file type is satisfactory before execution. In the future versions, we will implement a feature in Kronos to check the file types for all the components before executing the workflows.

* Since it was stated many times that heterogeneous compute environments can be used, is there any support for migrating the needed date between cloud and local clusters? Other workflow systems offer this.
Response: We assume that the reviewer meant “data” not “date”. If that is the case, users need to manually transfer data between cloud and local clusters. To our best knowledge, none of the well-know lightweight workflow management systems offer this as a feature.
If the reviewer did mean “date”, there is a mechanism for it, *i.e.* by using the sentinel files we can store timestamps and retrieve it later.

* parallelization of tasks is very important but how does this work for FASTA files or more complicated formats like bigwig or gtf? Is this easily extendable with the mentioned yaml file?
Response: We have implemented the parallelization mechanism as a combination of Kronos and components.
For each complicated file format, e.g. FASTA, FASTQ, there is a component to split that file, These components are made parallelizable by implementing a specific method of the *Component* class called *focus* method*.* In particular, the method instructs what input parameter of component should be used to parallelize and how the input arguments should pass to the subsequent child parameters. Kronos runs such components in parallel by making all the required child subtasks with proper input/output dependencies as well as passing proper input arguments to the component command via *focus* method.

The components need to be implemented only once. Therefore, all the splitting components can eventually become part of the Kronos installation.
For example, in one of our Kronos alignment workflows, we have a component for splitting FASTQ files and a component for running BWA. Both of these components are parallelizable. When running the workflow, the former splits the input FASTQ and Kronos performs the necessary actions for spawning and tracking the children subtasks. Once all the children successfully finish, Kronos spawns enough children for the latter component and passes the resulting smaller FASTQs to the corresponding children subtasks of the BWA task.

Unless a file format is intrinsically not splittable, any format can have a component for splitting that will enable parallel run on that file format.
This mechanism cannot be done from within the YAML configuration file. However, the parallelization can be switched on/off in the configuration file.
We will add this note to the official documentation.

* Describing Kronos as scheduler-agnostic is a little bit far fetched, I guess. Just using libdrmaa is not scheduler-agnostic and you need to normally do a lot more to get this right. For example can Kronos run on SLURM, Condor, Mesos, Moab ...?
Response: We agree. We have changed the text to eliminate the confusion (please see “Cloud

support" subsection). However, the support for different schedulers can be incorporated into the "job_manager" module of Kronos and in runtime accessed by the input option "-b".

General remarks:
* Reproducible research is more than defining and running workflows. It's about reproducible tool installations, annotation data, indices and the management thereof. This is really lacking in the manuscript if the authors aim to create a reproducible software platform.
* Workflows are easily transferable, but what about the Tools and associated data? This is a really important and hard part of reproducible pipelines.
Response: We agree with the reviewer's comment. Basically, workflows and components made by Kronos are portable. A workflow can be duplicated elsewhere using its configuration file.
Regarding the tools installation, given the nice capabilities of Docker, users can create a Docker image for each workflow that would contain all the required components and installations to facilitate the process of reproducibility of tools installation.
Regarding the associated data, Kronos cannot assist with its reproducibility due to the controlled accessibility and privacy policies around bioinformatics data. As stated before, users need to manually manage/transfer data whenever required.
We have amended the text to clarify this point (please see "Reproducible workflows" subsection).

* what is meant by the "build-in-runtime" manager?
Response: By "built-in run manager" we refer to the parts of the Kronos implementation that are responsible for parallelization/synchronization, job submission, making scripts, logging events, pause/resume of the workflow, and making run ID's.
We have amended the text to clarify this (please see page 4 first paragraph).

Spelling:
- toolshed -> Tool Shed
- "run either locally, on a cluster" -> or?
Response: Thank you. We have fixed them in the text.

Workflow management systems are very complicated and there are so many things, we as community, need to get right, that I don't expect Kronos to solve everything.
My main point is that Kronos claims to solve all this but, as I have hopefully shown, it does not, at least it's not written in the manuscript.
Even worse from what I have seen from looking at the code Kronos seems to be little more than sugar around Ruffus and this is not stated anywhere.
Response: As stated earlier, our intention is not to introduce a new workflow management system or to solve all the issues of other workflow systems. In our perspective, Kronos serves as the next step for the lightweight workflow management systems such as Ruffus and facilitates workflow development to a great extent.

I don't want to demotivate the authors and I think it's worth to spend time in this research area, but I always hope to solve this entire problem together and improve already existing technologies.
Response: We agree. We picked the existing workflow management system, Ruffus, and tried to take it to the next level.

If I'm right about my Ruffus assumption it would be great if this can be merged with Ruffus and made available as extension.
Sorry for this long review,

Response: We believe that Kronos is the missing frontend of lightweight workflow management systems like Ruffus. The backend of Kronos can be a different workflow system than Ruffus. So, Kronos has to be as a separate stand-alone tool.
We also contacted the developer of Ruffus to explore the possibility of merging Kronos with Ruffus and he also stated "there is a clear separation of concerns between Kronos and Ruffus". The focus of Kronos is to reduce the overhead of workflow development whereas Ruffus aims to eventually provide a GNU make-like python-based workflow management system.

Reviewer #2 (Brad Chapman): * Overview

The authors describe Kronos, a workflow management tool that encourages a standard way of defining analysis runs using configuration files as inputs.
Kronos is freely available with source code on GitHub, well documented, and installs easily using the Python pip installer. The usage of user editable top level configurations for running iterations on consistently re-run workflows is a nice addition to current practice. Below I provide suggestions on simplifying the presentation of Kronos usage and practice and defining how it fits with community standard development for representing workflows.
Response: We greatly appreciate your comments.

** Paper suggestions

- The presentation of using Kronos in the paper could use some re-organization to flow with usage. I was trying to work through a minimal example and had to jump around between seeds, components, the command lines to run and configuration file specifications. The documentation reads similarly and it's tough to follow a process like: do A, then B, then C. Having a worked example on a simple tool would be a big benefit to helping users go through the process.
Response: We have changed the text to fix this. However, due to the paper's structure restrictions we couldn't change it thoroughly.

- Kronos does require programming knowledge since creating components (wrappers for tools) requires editing a set of Python files. This is also acknowledged in the conclusions section of the paper but I feel like some of the arguments in the paper about not programming as a differentiating feature of Kronos should have a different presentation. Most users will probably not have a pre-existing set of components so will need some knowledge to do this. I appreciate the separation of workflows as a configuration-only input but also think some of the initial presentation sets up the wrong expectations.
Response: We agree. We have changed the text to clarify it more (please see "Abstract", "Kronos components" subsection and "Conclusions" section).

- I found the naming of components and seeds confusing. As far as I understand, components are wrappers for command line tools and typically referred to as tool wrappers in workflow languages. seeds appear to be the actual command line run.
It would be helpful to use more standard nomenclature for these.
Response: We replaced the term *seed* with *command line tool* to conform more to the standard nomenclature. However we retain the term *component* due to the difficulties in changing the naming convention at this stage.

- The paper mentions automatic parallelization and merge functionality, with the example of running a variant workflow in parallel over blocks of the genome. I could not find more details on this implementation in the kronos source. It would be helpful to expand on how this works, and

define the algorithms used to split up the tasks.
Response: We have implemented the parallelization mechanism as a combination of Kronos and components.
For instance, there is a component for splitting a specific file format, e.g. FASTA, FASTQ, etc. These components are made parallelizable by implementing a specific method of the *Component* class called *focus* method. In particular, the method instructs what input parameter of component should be used to parallelize and how the input arguments should pass to the subsequent child parameters. Kronos runs such components in parallel by making all the required child subtasks with proper inputs/outputs dependencies as well as passing proper input arguments to the component command via *focus* method.

For example, in one of our Kronos alignment workflows, we have a component for splitting FASTQ files and a component for running BWA. Both of these components are parallelizable, When running the workflow, the former splits the input FASTQ and Kronos performs the necessary actions for spawning and tracking the children subtasks. Once all the children successfully finish, Kronos spawns enough children for the latter component and passes the resulting smaller FASTQ's to the corresponding children subtasks of the BWA task.

In the process of parallelization, Kronos checks the component of the next task and if it can synchronize the two components then it would automatically parallelize the next component with proper inputs from the children of previous component too. This continues until the two components cannot be synchronized based on some specific criteria. In this case, the *merge* automatically happens before passing the files to the next component. The merge can support different type of files and we can add more types to it as we go forward. It can also be switched on/off in the configuration file.
We will add this note to the official documentation.

- The authors should mention the Common Workflow Language (http://www.commonwl.org/), a community effort to unify representation of workflow definitions and tool wrappers. As mentioned in the paper, there are currently a large number of existing workflow systems that cover this space and all argue to be the most user friendly and configurable, so movement towards a common standard for running analyses is a welcome change and I'd like to hear the authors' thoughts. There also may be other workflow systems worth mentioning in the background from this list:
https://github.com/common-workflow-language/common-workflow-language/wiki/Existing-Workflow-systems

Response: We have amended the text to address this comment (please see Introduction and Conclusions sections).

** Usage suggestions
- The set of example pipelines is very helpful for understanding Kronos. There is a link in the documentation:
http://kronos.readthedocs.org/en/latest/launch_pipeline/run.html#pipeline-repo
but it appears to point to a in-house repository without permissions to view.
This should point to the GitHub organization referenced in the paper:
https://github.com/MO-BCCRC?tab=repositories

Response: Thank you. We have fixed this now.

References

[1] Dobin, Alexander, et al. "STAR: ultrafast universal RNA-seq aligner." *Bioinformatics* 29.1 (2013): 15-21.

[2] STAR repository, https://github.com/alexdobin/STAR

[3] STAR manual, https://github.com/alexdobin/STAR/blob/master/doc/STARmanual.pdf
