# Supplementary material for: Kronos: a workflow assembler for genome analytics and informatics
Source: Gigascience. 2017 Jun 26;6(7):1–10. doi: 10.1093/gigascience/gix042 (PMC5569921; doi:10.1093/gigascience/gix042)

Kronos: a workflow assembler for genome analytics and informatics - *supplementary information*

We assume that the dependencies of `kronos` as well as pip are installed. Therefore, `kronos` can be installed using pip:

```
$ pip install --upgrade kronos-pipeliner
```

If the installation is successful, `kronos` would be available as a command. You can test it by:

```
$ kronos -v
```

which should print the version of `kronos`.

Also, you need to download the `strelka_workflow.tar.gz` tarball from our ftp server: `ftp://ftp.bcgsc.ca/public/shahlab/kronos`, and unpack it into a desired directory, say `$HOME_DIR`:

```
$ cd $HOME_DIR
$ tar -xvf strelka_workflow.tar.gz
```

This will make a directory called `strelka_workflow` in `$HOME_DIR`.

The test input data is a lightweight pair of cell line tumour/normal bam files that can be downloaded from here:

- Exome normal bam file
- Exome tumour bam file

Using this data, we will make plots similar to those in Figure 5 of the paper. We are using this lightweight pair of bam files so that this can be done on a local computer with decent amount of memory (>=8G). To reproduce the exact same plots in the Figure 5 of the paper, you need to download the data from EGA under the accession number EGAS00001000952 for the case *SA500* and run the workflow on a more powerful computer.

Section 1. Make a component

In this section, we are going to make a component named *plot_strelka* which is used to create Figure 5 of the manuscript. The *seed* of this component is an R script called `plot_strelka.R` which is included in the tarball under the `seeds` directory. It takes a Strelka result file as an input and generates a plot that contains the individual plots in Figure 5.

> ***Note***
>
> Refer to the online documentation for the definition of seed and component.

The seed can be run by the following command:

```
$ R --no-save --args <infile> <outfile_name> < plot_strelka.R
```

where `<infile>` and `<outfile_name>` should be replaced with a Strelka result file and a name for the output file, respectively.

We can make a component for this seed by following steps 1 to 6:

Step 1. Make a component template:

```
$ kronos make_component plot_strelka
```

This will make a component template called *plot_strelka* in the current working directory.

Step 2. Copy the seed to the `plot_strelka/component_seed` directory.

Step 3. Open the `plot_strelka/component_main.py` template file and add the following lines to the beginning of the `make_cmd` function:

```
cmd = self.requirements['R'] + ' --no-save --args '
cmd_args = [self.args.infile, self.args.outfile_name]
cmd_args.append('<')
cmd_args.append(os.path.join(self.seed_dir, 'plot_strelka.R'))
```

The above four lines are the only codes that we need to write for this component.

Essentially, the above four lines create the same command that is used to run the seed, i.e. `$ R --no-save --args <infile> <outfile_name> < plot_strelka.R`.

Generally, in the `component_main.py` of a component, we always only need to create the command that is used to run its seed.

For this component, we have chosen '`R --no-save --args`' as the command `cmd` in the first line, and the other three lines simply list the the rest of the command. Also, the following lines in the template file created by `kronos` are not required for this component and therefore can be deleted or commented out:

```
args = vars(self.args)
comp_seed_map = {
                 #e.g. 'component_param1': 'seedParam1',
                 #e.g. 'component_param2': 'seedParam2',
                }

for k, v in args.items():
    if v is None or v is False:
        continue

    ## TODO: uncomment the next line if you are using
    ## comp_seed_map dictionary.
    # k = comp_seed_map[k]

    cmd_args.append('--' + k)

    if isinstance(v, bool):
        continue
    if isinstance(v, str):
        v = repr(v)
    if isinstance(v, (list, tuple)):
        cmd_args.extend(v)
    else:
        cmd_args.extend([v])
```

Therefore the final `component_main.py` would look like:

```python
"""
component_main.py
This module contains Component class which extends
the ComponentAbstract class. It is the core of a component.

Note the places you need to change to make it work for you.
They are marked with keyword 'TODO'.
"""

from kronos.utils import ComponentAbstract
import os


class Component(ComponentAbstract):

    """
    TODO: add component doc here.
    """

    def __init__(self, component_name="plot_strelka",
                 component_parent_dir=None, seed_dir=None):

        ## TODO: pass the version of the component here.
        self.version = "v0.99.0"

        ## initialize ComponentAbstract
        super(Component, self).__init__(component_name,
                                        component_parent_dir, seed_dir)

    ## TODO: write the focus method if the component is parallelizable.
    ## Note that it should return cmd, cmd_args.
    def focus(self, cmd, cmd_args, chunk):
        pass
#         return cmd, cmd_args

    ## TODO: this method should make the command and command arguments
    ## used to run the component_seed via the command line. Note that
    ## it should return cmd, cmd_args.
    def make_cmd(self, chunk=None):
        ## TODO: replace 'comp_req' with the actual component
        ## requirement, e.g. 'python', 'java', etc.
        cmd = self.requirements['R'] + ' --no-save --args '
        cmd_args = [self.args.infile, self.args.outfile_name]
        cmd_args.append('<')
        cmd_args.append(os.path.join(self.seed_dir, 'plot_strelka.R'))

        if chunk is not None:
            cmd, cmd_args = self.focus(cmd, cmd_args, chunk)

        return cmd, cmd_args

## To run as stand alone
def _main():
    c = Component()
    c.args = component_ui.args
```

```
    c.run()

if __name__ == '__main__':
    import component_ui
    _main()
```

Step 4. As mentioned earlier, the seed takes as an input a Strelka result file and a name for the output file. We have chosen the names `infile` and `outfile_name` to represent these inputs, respectively. So, open the `plot_strelka/component_params.py` template file and add the names to it as follows:

```
"""
component_params.py

Note the places you need to change to make it work for you.
They are marked with keyword 'TODO'.
"""

## TODO: here goes the list of the input files. Use flags:
## '__REQUIRED__' to make it required
## '__FLAG__' to make it a flag or switch.
input_files  = {
                'infile' : '__REQUIRED__',
#                 'input_file2' : None
                }

## TODO: here goes the list of the output files.
output_files = {
                'outfile_name' : '__REQUIRED__',
#                 'output_file1' : None
                }

## TODO: here goes the list of the input parameters excluding input/output files.
input_params = {
#                 'input_param1' : '__REQUIRED__',
#                 'input_param2' : '__FLAG__',
#                 'input_param3' : None
                }

## TODO: here goes the return value of the component_seed.
## DO NOT USE, Not implemented yet!
return_value = []
```

> ***Note***
>
> You only need to change the following two lines:
>
> ```
> 'infile' : '__REQUIRED__',
> 'outfile_name' : '__REQUIRED__',
> ```

Step 5. Open the `plot_strelka/component_reqs.py` template file and only change the following line:

```
requirements = {
#                'python': '__REQUIRED__',
               }
```

to this:

```
requirements = {
                 'R': '__REQUIRED__',
               }
```

The rest of the fields in this file can also be changed if desired but is not required.

Step 6 (Optional). This step can be skipped. It is only needed if you want to run the component as standalone outside of a workflow. This step creates a user interface for the component. Open the `plot_strelka/component_ui.py` template file and change it so it looks like:

```
"""
component_ui.py

Note the places you need to change to make it work for you.
They are marked with keyword 'TODO'.
"""

import argparse

#===============================================================================
# make a UI
#===============================================================================
## TODO: pass the name of the component to the 'prog' parameter and a
## brief description of your component to the 'description' parameter.
parser = argparse.ArgumentParser(prog='plot_strelka',
                                 description = """
                                 creates a plot from Strelka results.""")

## TODO: create the list of input options here. Add as many as desired.
parser.add_argument(
                    "--infile",
                    default = None,
                    required = True,
                    help= """
                    input file.
                    """)

parser.add_argument(
                    "--outfile_name",
                    default = None,
                    required = True,
                    help= """
                    a name for the output file.
                    """)

## parse the argument parser.
args, unknown = parser.parse_known_args()
```

Section 2. Make a workflow

This section explains how to create the workflow used to generate the individual plots in Figure 5 of the manuscript. For this purpose, we need to use the components included in the `strelka_workflow.tar.gz` tarball. There are two components called `run_strelka` and `plot_strelka` in `$HOME_DIR/strelka_workflow/components` where `$HOME_DIR` is where you unpacked the tarball. You can also use the component we created in Section 1. Make a component for `plot_strelka`.

Next, export the components path to `PYTHONPATH` environment variable:

```
export PYTHONPATH=$HOME_DIR/strelka_workflow/components:$PYTHONPATH
```

Now, we can start making a new workflow using these component:

Step 1. Make a new configuration file using the `make_config` command:

```
kronos make_config run_strelka plot_strelka -o strelka_workflow
```

This will create a new configuration file called `strelka_workflow.yaml` in the current working directory. This file has a number of sections which we go through step by step. Please refer to the online documentation to learn more about the purpose of each section in the configuration file.

Step 2 (optional). The first section in the configuration file is `__PIPELINE_INFO__` and contains information regarding the workflow. Here is an example for this configuration file:

```
name: 'run_plot_strelka'
version: '1.0'
author: 'Jafar Taghiyar'
data_type: 'SNV'
input_type: 'bam'
output_type: 'vcf, jpeg'
host_cluster: 'local'
date_created: '2016-01-04'
date_last_updated:
Kronos_version: '2.0.4'
```

This section is only informative and does not have any effects on the workflow.

Step 3. The second section is `__GENERAL__` that lists the requirements of all the components in the workflow.

In this workflow, it looks like this:

```
strelka: '__REQUIRED__'
R: '__REQUIRED__'
perl: '__REQUIRED__'
```

These entries are required. However, these values can come from a setup file when running the workflow in Section 3. Run a workflow. So, a user does not need to pass values to them in the configuration file.

Step 4. The next section is `__SHARED__` where we can create variables.

In this workflow, we add the following variable to this section:

```
__SHARED__:
    strelka_ref: #a reference genome
```

Similar to __GENERAL__ section, the value for this entry can come from the setup file when running the workflow. In **Step 6**, we will see how we use it.

Step 5. Next is __SAMPLES__ section that can be used to list the input files or parameters. By default the section looks like:

```
__SAMPLES__:
    # sample_id:
    #    param1: value1
    #    param2: value2
```

In this workflow, the input files are a pair of tumour/normal bam files. Also, we choose a parameter from Strelka component called `min_tier2_mapq` to included as input in this section to show the functionality of the section.

The content of this section can be provided in an input file when running the workflow. So, a user does not need to pass values here. Please see Section 3. Run a workflow.

In **Step 6**, we will see how we use the content of this section.

Step 6. The rest of the configuration file contains __TASK__ sections. These sections are where the connections among different components in the workflow are specified. We also need to pass proper values to all the parameters in these sections that have __REQUIRED__ keyword as input. For example, in this workflow, we have the following entries with __REQUIRED__ as their values that we need to pass actual values to:

- in __TASK_1__ section:

```
tumour: __REQUIRED__
ref: __REQUIRED__
normal: __REQUIRED__
output_dir: __REQUIRED__
```

- in __TASK_2__ section:

```
infile: __REQUIRED__
outfile_name: __REQUIRED__
```

Some of these entries will be filled when specifying the flow of the workflow. For example, we like to get the input for the first task from an input file, *i.e.* from __SAMPLES__ section. For this purpose, we use sample connections:

```
__TASK_1__:
.
.
    component:
        input_files:
            tumour: ('__SAMPLES__', 'tumour')
            .
            .
            normal: ('__SAMPLES__', 'normal')
```

Next, we want the second task, __TASK_2__, to get its input from the output of the first task, __TASK_1__. Therefore, we simply pass the name of the output file from Strelka in the first task to the `infile` parameter of the second task:

```
__TASK_2__:
.
```

```
.
    component:
        input_files:
            infile: passed.somatic.snvs.vcf
```

and then we add the `__TASK_1__` to the `forced_dependecies` of `__TASK_2__` which makes `__TASK_2__` to wait for `__TASK_1__` to finish first:

```
__TASK_2__:
.
.
    run:
    .
    .
        forced_dependencies: ['__TASK_1__']
```

Note

Since Strelka software enforces the name of its result file to be *passed.somatic.snvs.vcf*, we need to use the exact same name in the configuration file and then use the `forced_dependencies`. Otherwise, `kronos` has a mechanism called IO-connection that can help automatically pass the output of one task to the input of another task. It also manages the dependencies automatically.

So far, We have already passed desired values to `tumour`, `normal`, and `infile`. For `output_dir` and `outfile_name` we only need to pick names. Let's choose `strelka_output` and `results/passed.somatic.snvs.pdf` for them, respectively:

```
__TASK_1__:
.
.
    component:
    .
    .
        output_files:
            output_dir: strelka_output
__TASK_2__:
.
.
    component:
    .
    .
        output_files:
            outfile_name: results/passed.somatic.snvs.pdf
```

> ### *Note*
>
> The `results/` in `results/passed.somatic.snvs.pdf` instructs `kronos` to make a directory called `results` and copy the result file `passed.somatic.snvs.pdf` there.

Since we want to enable a user to pass a reference genome in the setup file, *i.e.* without having to change the configuration file, we pass it as a variable in `__SHARED__` section (see **Step 4**). To use it, similar to sample connections, we use a shared connection:

```
__TASK_1__:
.
.
    component:
        input_files:
        .
        .
            ref: ('__SHARED__','strelka_ref')
```

All the connections will be automatically replaced in the runtime.

Step 7. In **Step 5**, we chose the `min_tier2_mapq` parameter to be included in the `__SAMPLES__` section. Therefore, we need to add a sample connection:

```
__TASK_1__:
.
.
    component:
        parameters:
        .
        .
            min_tier2_mapq: ('__SAMPLES__','mapq2')
```

> ### *Note*
>
> `mapq2` in the above line, is only an arbitrary key that we choose and it can be a different name. This key is used in the input file when running the workflow in Section 3. Run a workflow.

Rmember that this step is not required and we do it for education purposes.

The final configuraion file looks like this:

```
__PIPELINE_INFO__:
    name: 'run_plot_strelka'
    version: '1.0'
    author: 'Jafar Taghiyar'
    data_type: 'SNV'
    input_type: 'bam'
    output_type: 'vcf, jpeg'
    host_cluster: 'local'
```

```
    date_created: '2016-01-04'
    date_last_updated:
    Kronos_version: '2.0.4'
__GENERAL__:
    strelka: '__REQUIRED__'
    R: '__REQUIRED__'
    perl: '__REQUIRED__'
__SHARED__:
    strelka_ref: #a reference genome
__SAMPLES__:
    # sample_id:
    #    param1: value1
    #    param2: value2

__TASK_1__:
    run:
        # NOTE: component cannot run in parallel mode.
        boilerplate:
        num_cpus: 1
        memory: '10G'
        use_cluster: True
        add_breakpoint: False
        forced_dependencies: []
        env_vars:
    reserved:
        # do not change this section.
        seed_version: '1.0.13'
        component_version: '1.2.0'
        component_name: 'run_strelka'
    component:
        input_files:
            tumor: "('__SAMPLES__', 'tumour')"
            config:
            ref: "('__SHARED__','strelka_ref')"
            normal: "('__SAMPLES__', 'normal')"
        parameters:
            skip_depth_filters: "('__SHARED__','strelka_exome')"
            min_tier1_mapq: 20
            num_procs: 8
            min_tier2_mapq: "('__SAMPLES__','mapq2')"
        output_files:
            output_dir: 'strelka_output'
__TASK_2__:
    run:
        # NOTE: component cannot run in parallel mode.
        boilerplate:
        num_cpus: 1
        memory: '5G'
        use_cluster: True
        add_breakpoint: False
        forced_dependencies: ['__TASK_1__']
        env_vars:
    reserved:
        # do not change this section.
        seed_version: '0.99.0'
        component_version: '0.99.0'
```

```
        component_name: 'plot_strelka'
    component:
        input_files:
            infile: 'passed.somatic.snvs.vcf'
        parameters:
        output_files:
            outfile_name: 'results/passed.somatic.snvs.pdf'
```

Section 3. Run a workflow

In this section, we are going to run the simple tumour/normal pair single nucleotide variant calling workflow that we made in Section 2. Make a workflow. This workflow consists of two components and generates the plots in Figure 5 of the manuscript:

- *Component 1:* runs Strelka on a pair of tumour and normal `bam` files.
- *Component 2:* creates a series of plots from Strelka output.

Requirements

- Python >= v2.7.6
- Strelka == v1.0.14
- Java >= v1.7.0_06
- Perl >= v5.8.8+

How to run the workflow

Step 1. Create a setup.txt file:

```
#section key value
__GENERAL__ strelka <path to /strelka_install_dir/bin/configureStrelkaWorkflow.pl>
__GENERAL__ R <path to R executable, e.g. R>
__GENERAL__ perl <path to perl executable, e.g. perl>
__SHARED__ strelka_ref $HOME_DIR/strelka_workflow/refs/GRCh37.75.fa
```

> ***Note***
>
> The above file is a tab separated file and the first line, *i.e.* '`#section key value`', is part of the file. Also the *value* column should be replaced with actual values. Please note that a reference genome is included in the `strelka_workflow.tar.gz` tarball that can be used for `strelka_ref`. The `$HOME_DIR` should be replaced with the directory where the tarball is unpacked.

Step 2. Create an input.txt file:

```
#sample_id tumour normal mapq2
SAM_0 <tumour bam file path> <normal bam file path> 0
SAM_5 <tumour bam file path> <normal bam file path> 5
```

> ***Note***
>
> The above file is a tab separated file and the first line, *i.e.* '`#sample_id tumour normal mapq2`', is part of the file. `SAM_0` and `SAM_5` are arbitrary ID's. However, the ID's cannot be used more than once in an input file. For tumour and normal bam files, you can use either the lightweight pair of bam files or the EGA data mentioned in the begining of this document. If you already have a pair of bam files, then you can use them too. However, the plots will be different than what we present here.

Step 3. Run the workflow using the following command:

```
kronos run -c $HOME_DIR/strelka_workflow/components/ -e strelka
           -i input.txt -r TEST_RUN -s setup.txt -w RES
           -y <path to strelka_workflow.yaml> --no_prefix
```

Please note to replace `<path to strelka_workflow.yaml>` with the actual path.

Outputs

The resulting files will be saved in the directory current direcory under `RES` subdirectory. For this workflow, the final result files are:

- RES/TEST_RUN/SAM_0/outputs/results/passed.somatic.snvs.pdf
- RES/TEST_RUN/SAM_1/outputs/results/passed.somatic.snvs.pdf

Please refer to the online documentation for more information on the structure of the results directory.

If you have used the EGA data, then the resulting plots should be identical to the individual plots in Figure 5 of the paper. However, if you have used the lightweith pair of cell line data, then the plots should look like the following (NOTE: we have put all the resulting plots in one single plot for the sake of illustration):

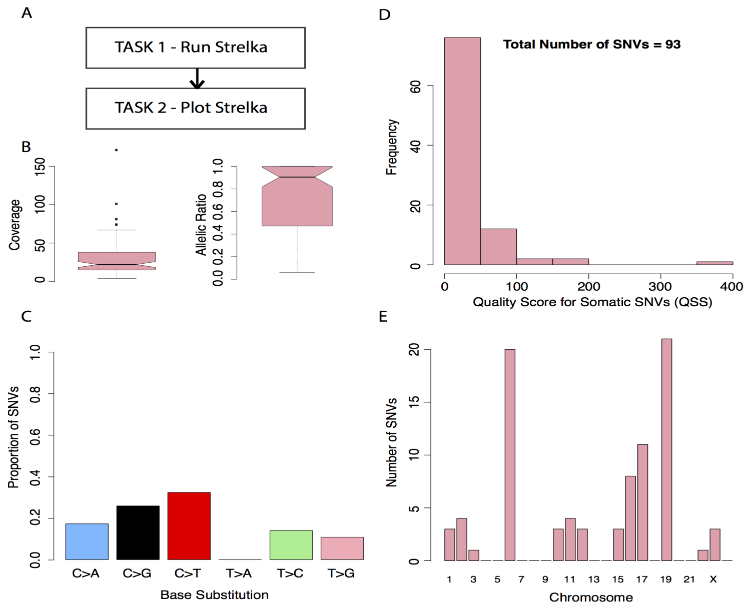

Section 4. Supplementary figures

Figure S1

Kronos configuration file has GENERAL, PIPELINE_INFO, SHARED, SAMPLES, and TASK's sections which are categorized as system-, user- and workflow-specific blocks. The PIPELINE_INFO section is merely informative and can be considered system-specific. The GENERAL section is system-specific and captures the system dependant requirements of the workflow such as the paths to the local installations. The SHARED and SAMPLES sections are user-specific and contain the input files and arguments. The TASK sections are workflow-specific and define the connection between the workflow *component*.

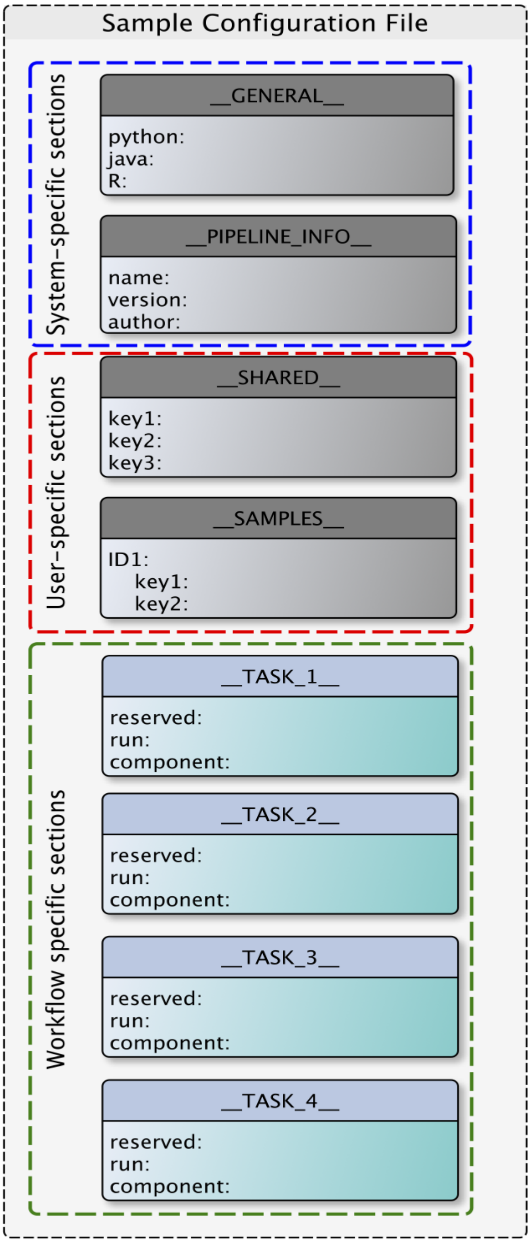

Figure S2

A task can be forced to wait for some other tasks to finish by simply passing the list of their names to the attribute `forced_dependencies` of the task. For example, in the sample configuration file in this figure, __TASK_8__ (GATK variant caller) is forced to wait for __TASK_6__ (the component that generates the bam file index) to finish first.

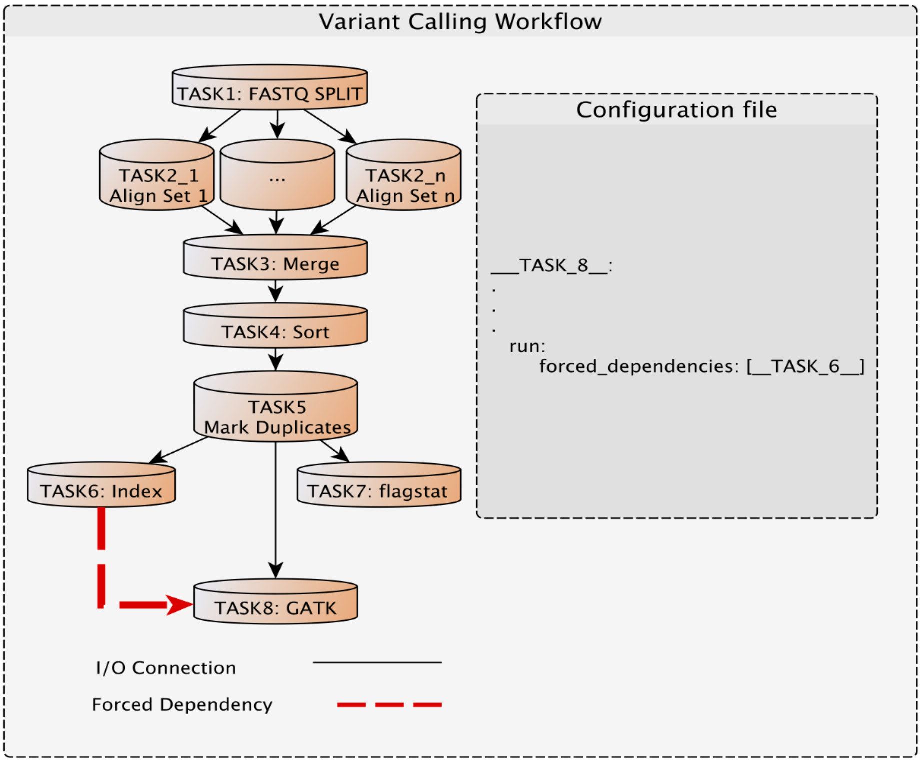

Supplement: Supplementary Information [file gix042_Supplementary-information.pdf]
