# Supplementary material for: Kronos: a workflow assembler for genome analytics and informatics
Source: Gigascience. 2017 Jun 26;6(7):1–10. doi: 10.1093/gigascience/gix042 (PMC5569921; doi:10.1093/gigascience/gix042)
Supplement: Response-to-Reviewer-Comments_Original-Submission.pdf [file gix042_Response-to-Reviewer-Comments_Original-Submission.pdf]

Author's Response To Reviewer Comments

Dear Dr. Edmunds,

As per your request, we have now edited the "Availability and requirements" section and also added the "Availability of Supporting Data" section to the manuscript.

Best Regards,
Sohrab Shah
